# TUBB3 and KIF21A in neurodevelopment and disease

**DOI:** 10.3389/fnins.2023.1226181

**Published:** 2023-08-04

**Authors:** Dharmendra Puri, Brenda J. Barry, Elizabeth C. Engle

**Affiliations:** ^1^Department of Neurology, Boston Children’s Hospital, Harvard Medical School, Boston, MA, United States; ^2^F. M. Kirby Neurobiology Center, Boston Children’s Hospital, Boston, MA, United States; ^3^Howard Hughes Medical Institute, Chevy Chase, MD, United States; ^4^Department of Ophthalmology, Boston Children’s Hospital, Harvard Medical School, Boston, MA, United States

**Keywords:** CFEOM, *KIF21A*, *TUBB3*, tublinopathy, tubulin, microtubule

## Abstract

Neuronal migration and axon growth and guidance require precise control of microtubule dynamics and microtubule-based cargo transport. *TUBB3* encodes the neuronal-specific β-tubulin isotype III, TUBB3, a component of neuronal microtubules expressed throughout the life of central and peripheral neurons. Human pathogenic *TUBB3* missense variants result in altered TUBB3 function and cause errors either in the growth and guidance of cranial and, to a lesser extent, central axons, or in cortical neuronal migration and organization, and rarely in both. Moreover, human pathogenic missense variants in *KIF21A*, which encodes an anterograde kinesin motor protein that interacts directly with microtubules, alter KIF21A function and cause errors in cranial axon growth and guidance that can phenocopy *TUBB3* variants. Here, we review reported *TUBB3* and *KIF21A* variants, resulting phenotypes, and corresponding functional studies of both wildtype and mutant proteins. We summarize the evidence that, *in vitro* and in mouse models, loss-of-function and missense variants can alter microtubule dynamics and microtubule-kinesin interactions. Lastly, we highlight additional studies that might contribute to our understanding of the relationship between specific tubulin isotypes and specific kinesin motor proteins in health and disease.

## Introduction

### Human missense variants in *TUBB3* and *KIF21A* cause errors in neuronal development

In 2010, human heterozygous *TUBB3* missense variants were reported to cause isolated and syndromic congenital fibrosis of the extraocular muscles (CFEOM), a congenital paralytic eye movement disorder (Tischfield et al., 2010). CFEOM is one of a series of rare Mendelian disorders that result from errors in cranial motor neuron development and are referred to as the “congenital cranial dysinnervation disorders” or CCDDs (Gutowski et al., 2003). CFEOM is an ocular CCDD defined by eyelid ptosis and non-progressive paralytic strabismus with restricted upgaze and variably restricted down and horizontal eye movements, with or without aberrant residual eye movements. Notably, the eyes cannot be moved actively or passively into restricted positions of gaze. Autosomal dominant (AD) CFEOM results from errors in extraocular muscle (EOM) innervation by the oculomotor nerve (cranial nerve III; CN III) and, in some individuals, also the abducens nerve (CN VI). Magnetic resonance imaging (MRI) and postmortem examination of individuals with CFEOM reveal hypoplasia of the oculomotor nerve with failure of its superior division to properly innervate its normal targets, resulting in hypoplasia of the levator palpebrae superioris and the superior rectus muscles and in ptosis and restricted upgaze. There can also be failure of its inferior division and abducens nerves to target their muscles, resulting in restricted down- and horizontal gaze (Engle et al., 1997; Demer et al., 2005, 2010; Tischfield et al., 2010). While CFEOM does not progress, affected individuals can develop amblyopia, secondary corneal damage, and upper back and neck discomfort secondary to aberrant head positioning.

**Figure 1:**
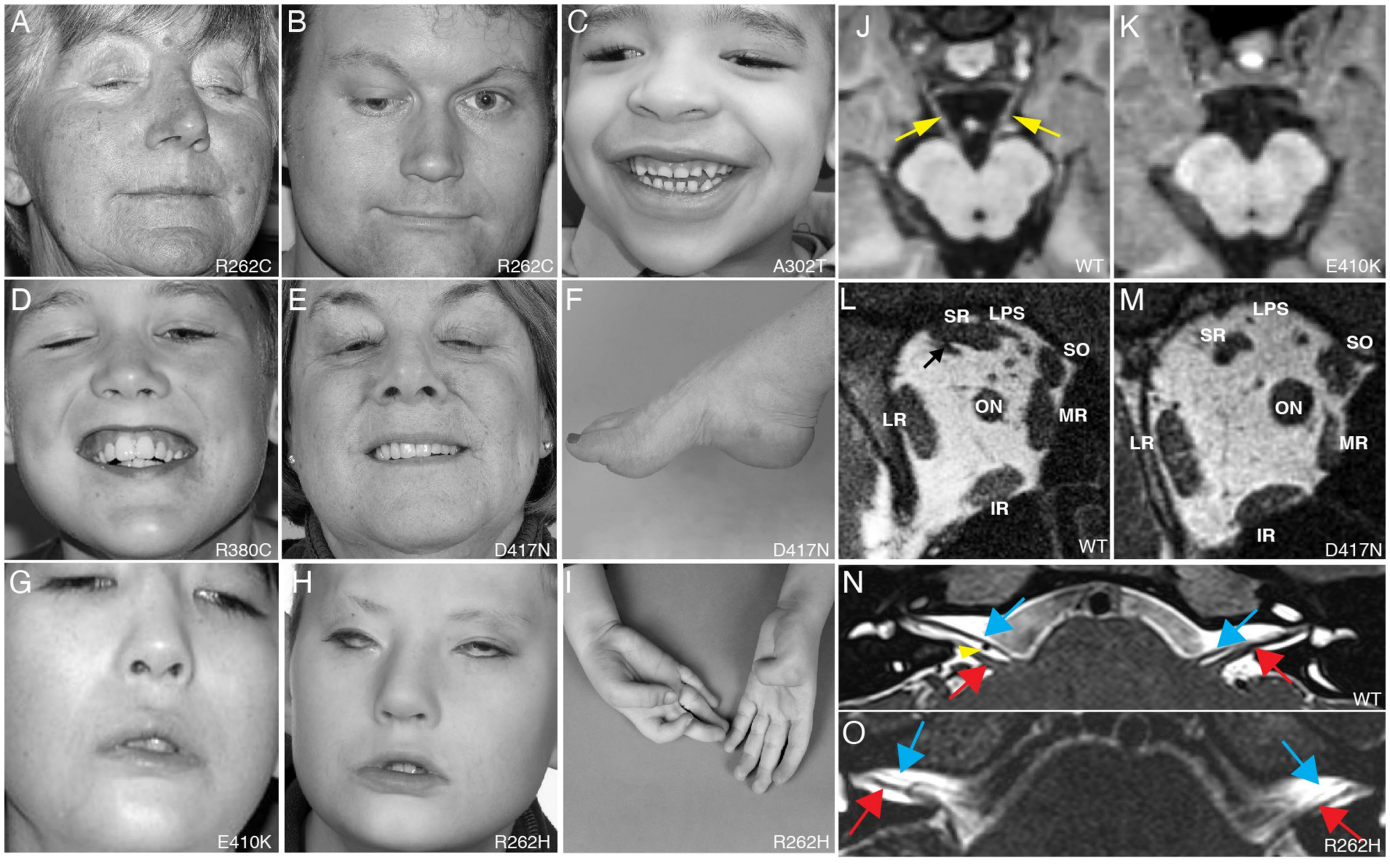
Clinical spectrum and cranial nerve imaging of the TUBB3-CFEOM syndromes. **(A–I)** Photographs of individuals harboring TUBB3-CFEOM variants. R262C can cause bilateral ptosis and severe CFEOM3 with the resting position of both eyes infraducted and abducted **(A)**, moderate CFEOM3 that can be unilateral **(B)**, and mild CFEOM3 (not shown). A similar spectrum is seen with D417N; severe CFEOM3 is shown in **(E)**. A302T **(C)** and R380C **(D)** cause moderate to severe CFEOM3. Participants in **(A–E)** have full facial movements. The axonal neuropathy in the participant with D417N **(E)** results in atrophy of the intrinsic foot muscles and a high arch **(F)**. E410K **(G)** and R262H **(H)** result in severe CFEOM3 and facial weakness, and R262H also results in congenital joint contractures, including ulnar deviation of the hand with joint contractures of the thumbs and fingers **(I)**. **(J,K)** MRI of the brainstem at the level of the oculomotor (CN III) nerve in control **(J)** and E410K **(K)**. **(L,M)** Orbital contents posterior to the globe in control **(L)** and in an individual with the E410K substitution. Note atrophy of the levator palpebrae superioris (LPS), superior rectus (SR), and medial rectus (MR) muscles in (M). The inferior rectus (IR), lateral rectus (LR), and superior oblique (SO) muscles appear normal. ON denotes optic nerve. **(N,O)** MRI of the brainstem at the level of the superior internal auditory meati to visualize the facial (CN VII, blue arrows) and vestibulocochlear (CN VIII, red arrows) nerves. In a control individual **(N)**, CN VII courses parallel and ventral to CN VIII on each side. The anterior inferior cerebellar artery flow void is seen between the cranial nerves on the right (yellow arrowhead). In an individual with R262H substitution, CN VII appears hypoplastic and is faintly visualized coursing ventral and parallel to the cranial nerves VIII. **(A–L)** Reproduced with permission from Tischfield et al. (2010); **(N,O)** reproduced with permission from Whitman et al. (2021).

**Figure 2:**
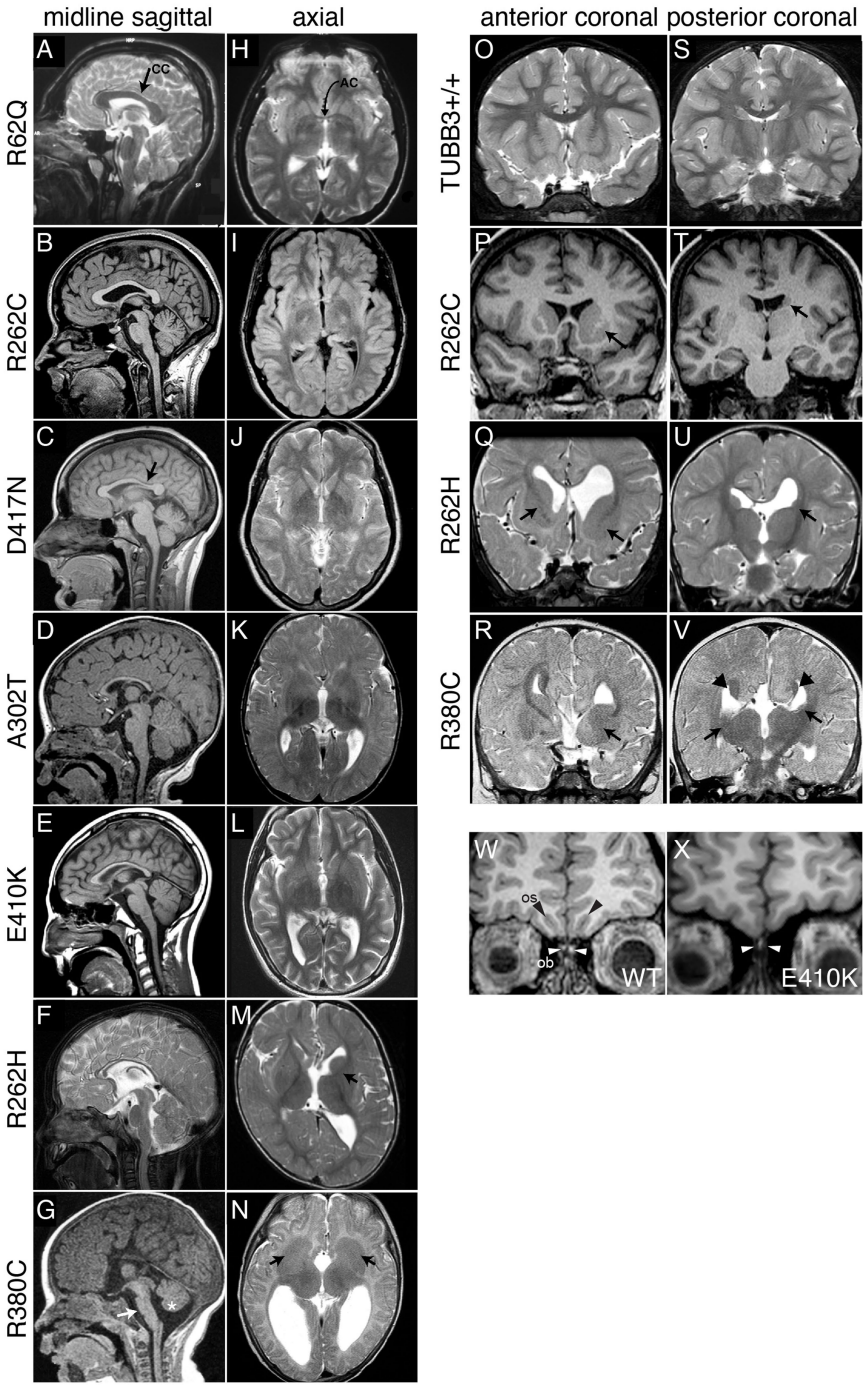
Spectrum of TUBB3-CFEOM brain malformations correlates with specific *TUBB3* variants. **(A–G)** Midline sagittal MRI showing the spectrum of corpus callosum (CC) dysgenesis; corresponding amino acid substitutions are noted to the left. R62Q **(A)** and most R262C **(B)** subjects have normal corpus callosum development, whereas D417N subjects have hypoplasia of the posterior body (**C**, arrow). Subjects with A302T, E410K, and R262H have diffuse corpus callosum hypoplasia **(D–F)**. **(G)** R380C subjects can have corpus callosal agenesis and brainstem (arrow) and mild vermian hypoplasia (asterisk). **(H–N)** Axial MRI from the same subjects’ scans showing the spectrum of anterior commissure (AC) dysgenesis and overall loss of white matter compared to the normal R62Q scan (**H**, arrow indicates AC). **(I–L)** Subjects have hypoplastic AC. R262H **(M)** and R380C **(N)** subjects have anterior commissure agenesis and dysmorphic basal ganglia. The anterior limb of the left internal capsule is hypoplastic in R262H (**M**, arrow), whereas there is lack of clear separation between the caudate and putamen and bilateral hypoplasia of the anterior limbs of the internal capsule with R380C (**N**, arrows). **(O–V)** Anterior **(O–R)** and posterior **(S–V)** coronal sections showing the spectrum of basal ganglia dysmorphisms. Compared to a TUBB3+/+ scan **(O,S)**, the R262C scan reveals asymmetric basal ganglia with enlargement of the left caudate head and putamen (**P**, arrow) and hypoplasia of the left caudate body (**T**, arrows). The R262H scan reveals dysgenesis of the left and right anterior limbs of the internal capsule (**Q**, arrows), apparent fusion of an enlarged left caudate head with the putamen **(Q)**, hypoplasia of the left caudate body and tail **(U)**, and asymmetrical dilatation of the lateral ventricles. The R380C scan reveals hypoplasia of the anterior limb of the internal capsule (**R**, arrow), fusion of the left caudate head and underlying putamen with bilateral hypoplasia of the caudate body and tail (**V**, arrows), and Probst bundles of callosal axons that line the bodies of the lateral ventricles (**V**, arrowheads). **(W,X)** Coronal images of olfactory sucli (OS, black arrows) and bulbs (OB, white arrows) in a control **(W)** compared to a subject with the E410K substitution **(X)**. The E410K subject has olfactory sulcus agenesis and bulb dysgenesis (**X**, white arrows). **(A–V)** Reproduced with permission from Tischfield et al. (2010). **(W,X)** Reproduced with permission from Chew et al. (2013).

While all affected individuals with the initial report of *TUBB3* variants had CFEOM, a subset of missense variants resulted in various combinations of additional clinical and magetic resonance imaging (MRI) findings, including additional CN dysfunction, progressive axonal motor-sensory polyneuropathy, intellectual disabilities, social impairments/autism spectrum disorder (ASD), congenital joint contractures, cyclic vomiting and cardiac arrhythmias. MR imaging also revealed variant-specific changes in peripheral and central white matter, including hypoplasia of CNs, anterior commissure, and corpus callosum, as well as malformed basal ganglia and thalami in the absence of cortical malformations (Demer et al., 2010; Tischfield et al., 2010; Chew et al., 2013; Whitman et al., 2021; Figures 1, 2).

Intriguingly, heterozygous missense variants in *KIF21A*, which encodes an anterograde kinesin motor protein (Marszalek et al., 1999), were reported in 2003 to cause isolated CFEOM (Yamada et al., 2003; Cheng et al., 2014) and the phenotypic overlap between TUBB3-CFEOM and KIF21A-CFEOM suggested a shared disease mechanism underlying these two disorders.

**Figure 3:**
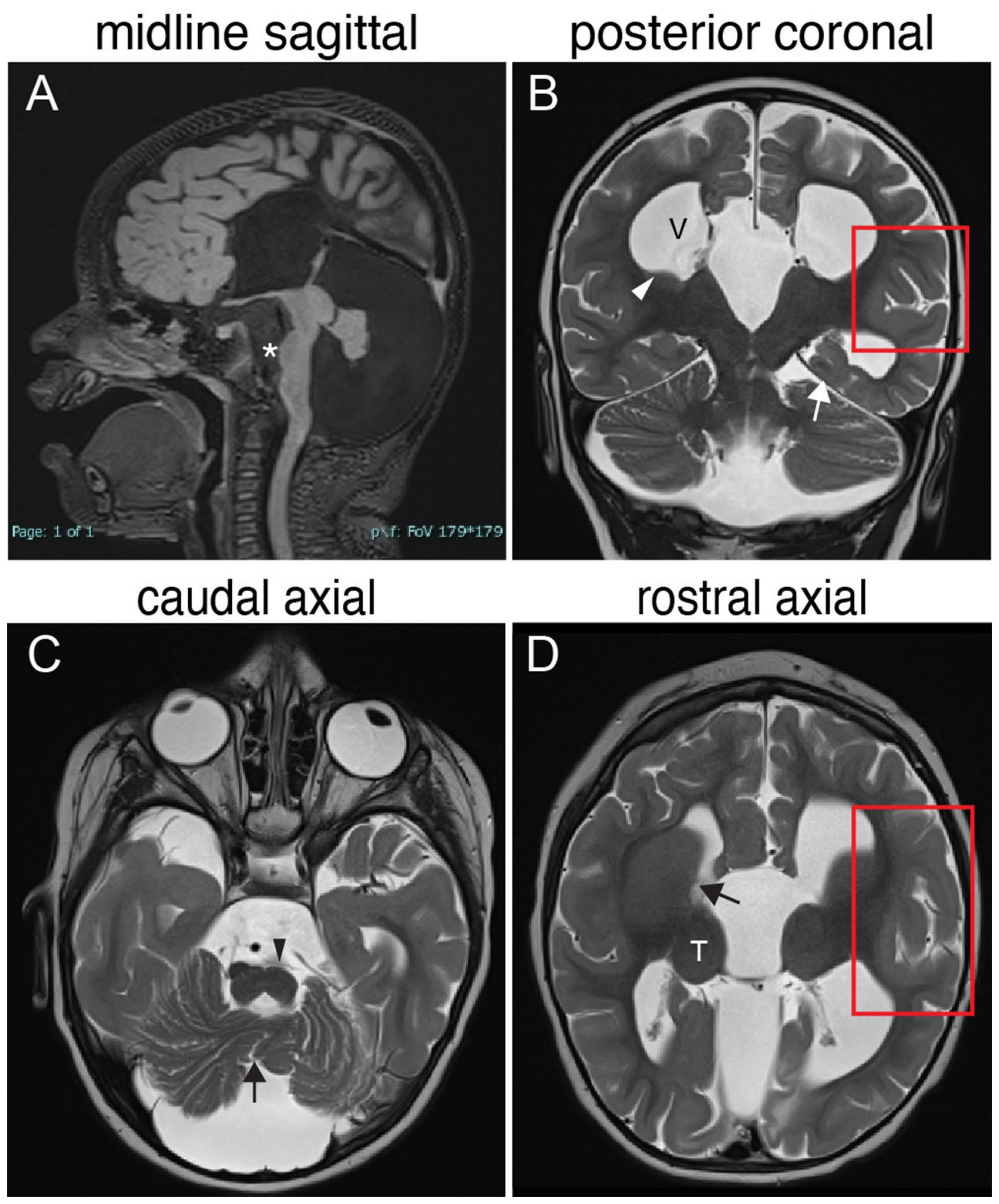
TUBB3-MCD brain malformations in individual harboring a TUBB3 T178M substitution. 17-month-old born at term presented with microcephaly, global developmental delay, axial hypotonia, appendicular hypertonia, and infantile spasms and underwent MR imaging. **(A)** Midline sagittal MR image: agenesis of the corpus callosum and anterior commissure, dysmorphic dilated ventricles, uplifted hypoplastic cerebellar vermis with enlarged CSF spaces, effaced aqueduct, malformed brainstem with flattened pons (asterisk). **(B)** Posterior coronal MR image: enlarged ventricles (V), bilateral hypoplasia of the caudate body and tail (white arrowhead), abnormal under-rotated left hippocampus (white arrow), abnormally oriented cerebellar folia, and thickened malformed cortex in the left Sylvian fissure (red boxed region). **(C)** Caudal axial MR image: hypoplastic malformed cerebellar vermis (black arrow) and hemispheres, flatted left pyramidal track (black arrowhead) and enlarged CFS spaces. **(D)** Rostral axial MR image: malformation of the basal ganglia with fusion of the caudate and putamen with no visible anterior limb of the internal capsule (black arrow), rounded globular-appearing thalami (T), ventricular enlargement, and thickened malformed cortex around the left Sylvian fissure (red boxed region).

A second 2010 report of heterozygous, often *de novo*, *TUBB3* missense variants expanded the phenotypic spectrum of TUBB3 amino acid substitutions to include subjects without CFEOM who, instead, had brain neuroimaging described as showing malformations of cortical development (MCD) (Figure 3). MCDs are a heterogenous group of malformations that result from errors in cortical neuronal proliferation, migration, or postmigrational development. While they can be diagnosed by MRI, the ability to recognize specific MCD subtypes can be impacted by imaging modality, technical resolution, and the subject’s age (Barkovich et al., 2019). Moreover, MCD classification has evolved over time (Severino et al., 2020). As a result, MCD nomenclature can be variable and inconsistent, making it challenging in some instances to assign definitive terminology to TUBB3-MCDs (Bahi-Buisson et al., 2014). That said, TUBB3-MCD variants are reported to cause a spectrum of cortical malformations, including simplified gyral patterns, dysgyria, polymicrogyria, and lissencephaly but which are, overall, less severe than MCDs resulting from *TUBA1A* or *TUBB2B* variants (Bahi-Buisson et al., 2014). In particular, dysgryia is frequently reported; this term has been used specifically in relation to tubulin pathogenic variants to describe a cortex of normal thickness that has an atypical pattern of cortical gyration with abnormally oriented and located sulci of varying depths (Mutch et al., 2016). While dysgyria is classified as an MCD, it is possible that it is not primary but instead arises secondarily to abnormal white matter development. Consistent with milder findings by MRI, while TUBB3-MCDs can result in intellectual disabilities, ASD, and seizures (Poirier et al., 2010), some individuals have very mild phenotypes as described below.

Following these initial reports of TUBB3-CFEOM, KIF21A-CFEOM, and TUBB3-MCD, the phenotypic spectrum and depth of understanding of human conditions associated with pathogenic or presumed pathogenic variants in *TUBB3* and *KIF21A* continue to expand. As detailed below, this includes rare *KIF21A* missense variants reported to cause not only CFEOM, but also facial weakness with or without progressive axonal motor-sensory polyneuropathy and intellectual/social disabilities (Ali et al., 2013; Soliani et al., 2020; Jia et al., 2022) increasing the overlap between KIF21A-CFEOM and TUBB3-CFEOM phenotypes. Here, we introduce TUBB3 and KIF21A function in health, review the reported human *TUBB3* and *KIF21A* variants and their corresponding phenotypes, and discuss the current understanding of the roles of TUBB3 and KIF21A in these developmental disorders.

### Microtubules and the TUBB3 β-tubulin isotype

Neuronal development and maintenance is regulated by intracellular and extracellular factors (Yogev and Shen, 2017), and a pivotal role is played by the intracellular microtubule cytoskeleton (Witte et al., 2008; Conde and Cáceres, 2009). Microtubules provide structure to the neuron, generate cellular forces, provide tracks for intracellular transport by kinesins and dynein, and serve as anchors for organelles and as a component of signaling pathways (Akhmanova and Steinmetz, 2008; Fletcher and Mullins, 2010; Hirokawa et al., 2010; Forges et al., 2012; Maday et al., 2014). Microtubules are assembled from tubulin heterodimers of different α- and β-tubulin isotypes. These tubulin heterodimers come together in a head-to-tail fashion to form polar protofilaments and the protofilaments then associate laterally to form hollow, cylindrical microtubules (Nogales, 2001). Microtubules frequently transition between states of polymerization (growth) and depolymerization (shortening), an intrinsic behavior referred to as “dynamic instability” (Mitchison and Kirschner, 1984). Microtubule organization and dynamic remodeling is critical to neuronal function, and is regulated by free tubulin concentration (Mitchison and Kirschner, 1984; Desai and Mitchison, 1997), cell-type-specific spatial and temporal regulation of tubulin isotype expression (Panda et al., 1994; Nogales, 2001), regulation of protofilament number (Janke and Kneussel, 2010; Chaaban and Brouhard, 2017; Roll-Mecak, 2020), binding of molecular motors and microtubule associated proteins (MAPs) (Desai et al., 1999; Hendershott and Vale, 2014), and posttranslational tubulin modifications (Janke and Magiera, 2020).

**Figure 4:**
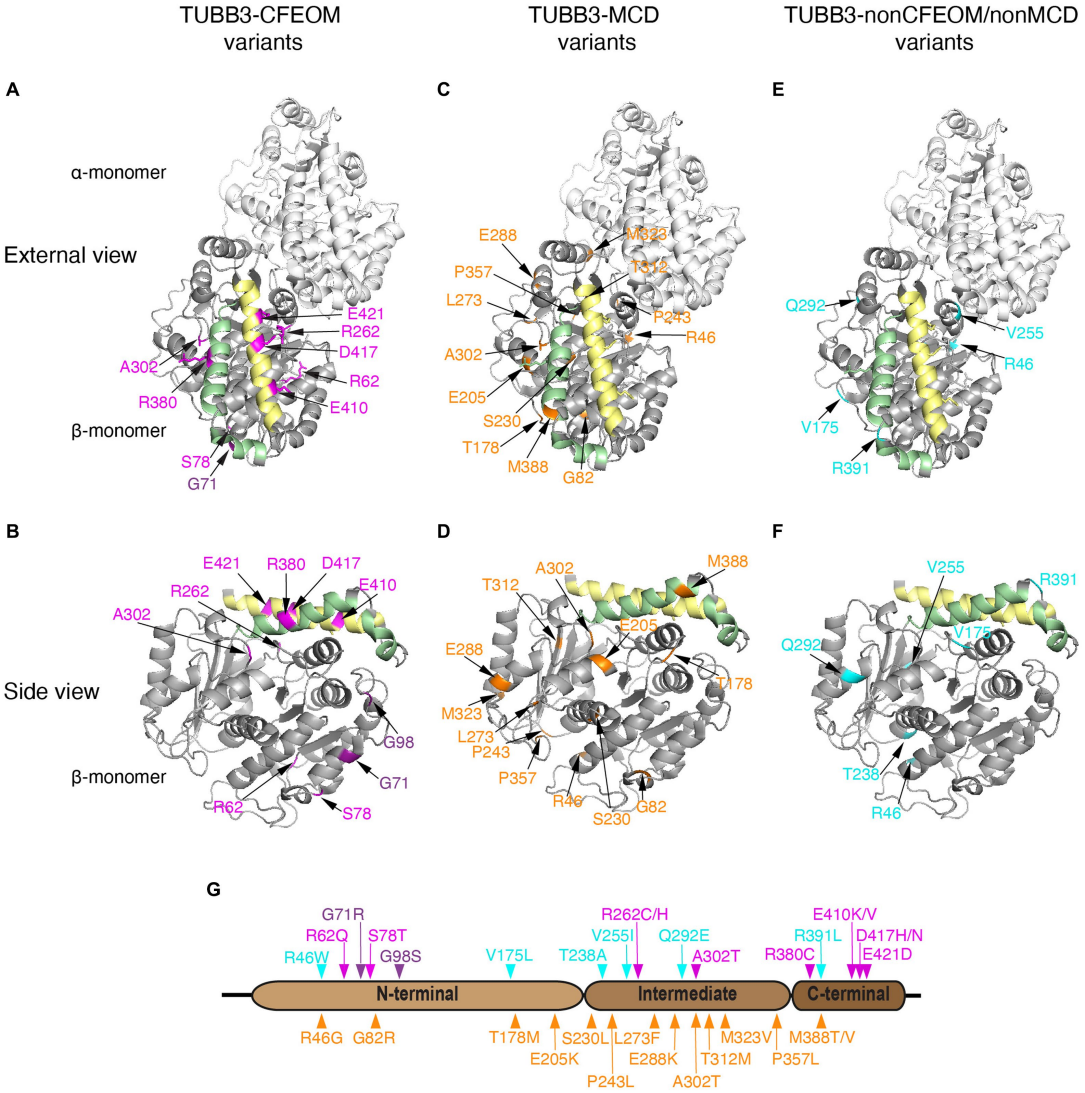
Human TUBB3 amino acid substitutions. **(A–F)** Three-dimensional schematics of TUBB3 structure with locations of reported TUBB3 human substitutions highlighted generated using PyMol (https://www.rcsb.org/structure/1JFF) and rotated to reveal: ‘external views’ (row 1; **A,C,E** displayed as heterodimers with α-tubulin) and ‘side views’ (row 2: **B,D,F**, displayed as β-tubulin monomers) highlighting helices H11 (green) and H12 (yellow) on the outside of the cylindrical hollow microtubule. TUBB3-CFEOM substitutions are highlighted in magenta and TUBB3-CFEOM/MCD in purple **(A,B)**. These substitutions are most often found in the C-terminal domain on or adjacent to helix H12 (yellow) on residues where motor protein interact. The exceptions are: R380 on helix H11 (green) and A302 that may interact with H11; R62 located in a loop mediating lateral interactions; and S78 together with TUBB3-CFEOM/MCD substitutions at G71 and G98 which cluster together and away from other variants near the E site of the GTP binding pocket. TUBB3-MCD substitutions are highlighted in orange **(C,D)**. These substitutions are located primarily in regions that regulate GTP binding, heterodimer stability, and longitudinal and lateral interactions and away from variants in **(A,B)**. Exceptions are A302 which is altered to a different residue in TUBB3-CFEOM and is located within a loop that could be important for both heterodimer stability and MAP/motor protein interactions. Similarly, M388 could regulate MAP/motor protein interactions, and is in proximity to residues at the plus-end of β-tubulin that mediate inter heterodimer contacts. TUBB3-nonCFEOM/nonMCD substitutions are highlighted in turquois **(E,F)**. These substitutions are also removed from H11/H12 helices and are located in regions that regulate heterodimer stability and longitudinal and lateral interactions. Refer to Tischfield et al. (2011) for 3D domain schematics. **(G)** Two-dimensional schematic of TUBB3 N-terminal, intermediate, and C-terminal domains with human amino acid substitutions indicated. TUBB3-CFEOM (magenta), TUBB3-CFEOM/MCD (purple), and TUBB3-nonCFEOM/nonMCD (cyan) amino acid substitutions are shown above, while TUBB3-MCD amino acid substitutions (orange) are shown below the TUBB3 protein schematic.

Mouse and human genomes encode at least eight α-tubulin and eight β-tubulin isotypes (Khodiyar et al., 2007; Redeker, 2010; Hausrat et al., 2021). Each isotype is comprised of an N-terminal, intermediate, and C-terminal domain. The N-terminus contains a GTP binding site necessary for tubulin folding, heterodimer stability, and microtubule dynamics. The intermediate domain mediates longitudinal and lateral interactions necessary for both tubulin heterodimer and microtubule stability (Löwe et al., 2001; Nogales and Wang, 2006). The C-terminal domain forms two parallel alpha helices on the external surface of tubulin where kinesin and dynein motor proteins (helix H12) and MAPs (helix H11) interact (Löwe et al., 2001; Kikkawa and Hirokawa, 2006; Nogales and Wang, 2006; Uchimura et al., 2006) and contains a C-terminal tail that is highly divergent among tubulin isotypes and is a site of post-translational modifications (PTMs) (Figure 4). In β-tubulin, the N-terminal, intermediate, and C-terminal domains correspond to amino acid residues 1–229, 230–371, and 372–450, respectively (Löwe et al., 2001). The increase in isotype diversity with an increase in species complexity, coupled with the differential spatial and temporal expression of specific tubulin isotypes, led to the belief that distinct tubulin isotypes evolved to diversify microtubule function within and between cell types (Sullivan et al., 1986; Luduena, 1993).

Within the nervous system, *TUBB3* is expressed only in neurons, where it can be visualized throughout the soma and processes of all post-mitotic neurons in the central and peripheral nervous system *in vivo* (Lee et al., 1990; Jiang and Oblinger, 1992; Menezes and Luskin, 1994; Dráberová et al., 2008) and *in vitro* (Ferreira and Caceres, 1992; Menezes and Luskin, 1994; Guo et al., 2011; Huang et al., 2015; Hausrat et al., 2021). It is also reported to be expressed in fibroblasts (Poirier et al., 2010) and in Sertoli cells of the testes (Dráberová et al., 1998; Gendt et al., 2011), and is upregulated in avian pre-migratory cranial neural crest prior to cell delamination and migration (Chacon and Rogers, 2019) and overexpressed in some tumors where it is a marker of poor prognosis (McCarroll et al., 2010).

In mice, *Tubb3* expression begins around embryonic day 9 (~E9) in the earliest-born neurons; expression is highest during development, where it is estimated to constitute ~20–40% of β-tubulin protein in mice (Latremoliere et al., 2018), but continues throughout the life of the neuron (Easter et al., 1993; Liu et al., 2007; Miller et al., 2014; Hausrat et al., 2021; Park et al., 2021). Expression also increases in mature neurons following neuronal activity (Radwitz et al., 2022) or injury (Moskowitz and Oblinger, 1995). In addition to TUBB3, neuronal microtubules are particularly enriched in α-tubulins TUBA1A (Zhang et al., 2014; Aiken et al., 2017), and β-tubulin TUBB2B (Banerjee et al., 1988); by real-time qPCR, the relative level of *Tubb3* expression is equal to or greater than *Tubb2b* (Braun et al., 2010). In the adult mouse brain, *Tubb3* expression levels vary somewhat by anatomic location, being higher in brainstem and striatum compared to cortex, hippocampus, olfactory bulb, and cerebellum (Braun et al., 2010). The TUBB3 specific monoclonal antibody, TuJ1, serves as a marker of neurons from early differentiation onward (Lee et al., 1990; Easter et al., 1993). As reviewed below, TUBB3 increases microtubule dynamics and may contribute to the formation of canonical thirteen-protofilament microtubules.

### KIF21A is an anterograde kinesin that regulates microtubule dynamics

**Figure 5:**
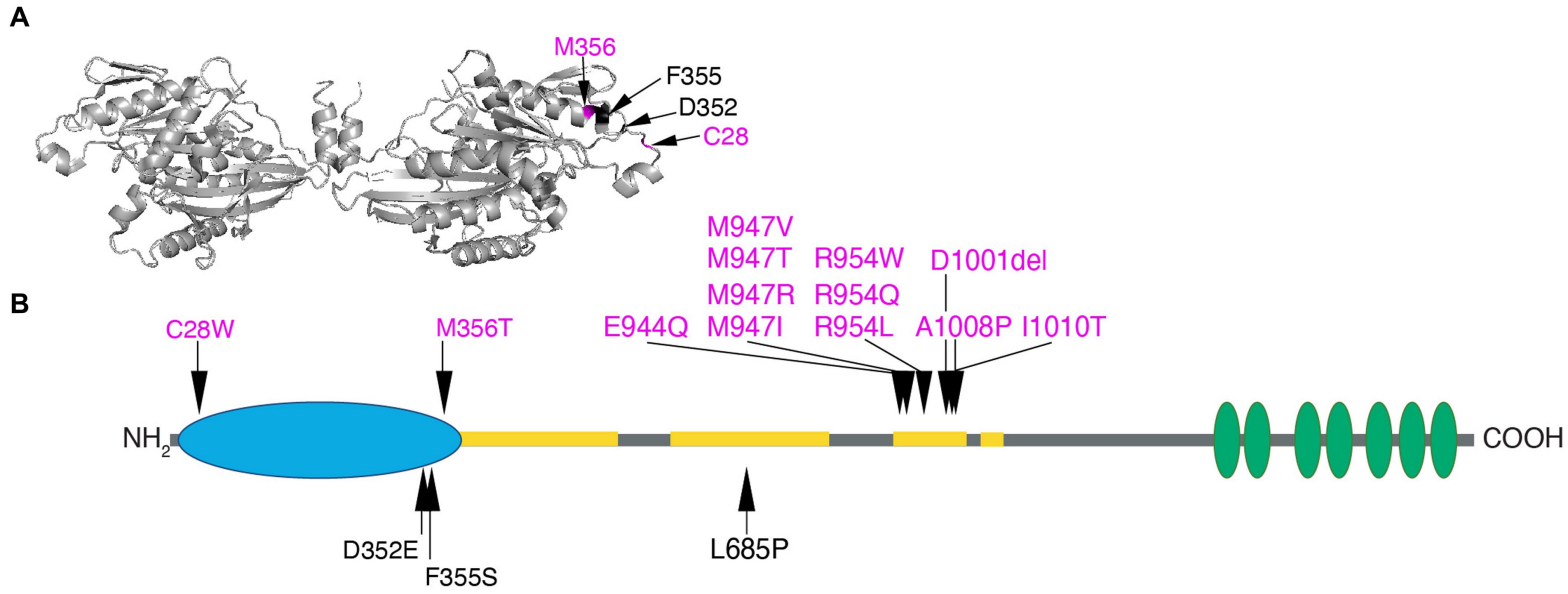
Human KIF21A amino acid substitutions. **(A)** Three-dimensional schematic of the *D. melanogaster* kinesin-1 motor domain dimer (https://www.rcsb.org/structure/2Y5W) with the four human variants that alter residues in the motor domain mapped to the monomer on the right. C28 and M345 that cause isolated CFEOM are denoted in magenta and D352 and F355 that cause more syndromic CFEOM are denoted in black. The variants cluster on the lateral region of the motor domain removed from the ATP and microtubule binding sites. Data supports this as the site of the motor-third coiled-coil stalk domain interaction for KIF21A autoinhibition. **(B)** Two-dimensional schematic of KIF21A motor (blue), stalk (gray) and WD40 (green) domains. The coiled-coil regions of the stalk are denoted in yellow. Substitutions that cause isolated CFEOM are denoted in magenta above the KIF21A schematic. In addition to the two motor substitutions, note the clustering of KIF21A-CFEOM substitutions in the third coiled-coil region of the stalk. This region has been demonstrated to interact with the lateral aspect of the motor domain. The three substitutions that cause more syndromic CFEOM are noted in black below the KIF21A schematic. In addition to the two in the distal motor domain, L685P maps to the second coiled-coil and results in a syndromic phenotype similar to the E410K TUBB3 syndrome.

*KIF21A* encodes a widely expressed kinesin motor protein, KIF21A, present in the soma and all processes but concentrated in the axon of developing and mature neurons of both the central and peripheral nervous system (Marszalek et al., 1999; Desai et al., 2012; Huang and Banker, 2012). KIF21A and KIF21B, which share ~65% amino acid identity, are plus-end directed N-kinesins with N-terminal motor domains, intermediate stalk domains containing coiled-coil motifs, and are distinguished from other kinesins by their C-terminal WD40 domains (Marszalek et al., 1999; Hirokawa et al., 2009; Figure 5). When not bound to the microtubule, KIF21A is autoinhibited through interaction of the stalk third coiled-coil with the motor domain (van der Vaart et al., 2013; Cheng et al., 2014; Bianchi et al., 2016). When associated with the microtubule, KIF21A is reported to participate in both cargo transport and microtubule dynamics. In rat hippocampal neurons, it was reported to transport the potassium-dependent sodium-calcium exchanger 2 protein (NCKX2) through an interaction with its WD40 domain, and KIF21A knockdown caused calcium dysregulation at axonal boutons *in vitro* (Lee et al., 2012). The importance of KIF21A cargo transport *in vivo*, however, remains unclear. KIF21A’s role in regulation of microtubule dynamics is better established; *Kif21a* overexpression *in vitro* and in dorsal root ganglia (DRG) neurons suppressed microtubule growth and catastrophe (the transition from polymerization to depolymerization), while *KIF21A* knockdown in HeLa cells led to increased microtubule growth (van der Vaart et al., 2013). KLP-12, the *C. elegans* homolog of KIF21A/B, interacted with β-tubulin E410 and inhibited growth and catastrophe of microtubules (Taguchi et al., 2022). The role of KIF21A in the control of microtubule dynamics at the cell cortex through the interaction of its stalk with the KN motif and ankyrin repeat domains 1 protein (KANK1) is discussed below relative to the mechanism underlying KIF21A amino acid substitutions in CFEOM.

## Human *TUBB3* variants

### *TUBB3* and *KIF21A* reference sequences

When assessing human genes and variants in the literature and clinical setting, it is critical to note the reference gene and protein used and, when possible, to follow standardized nomenclature. This is particularly important for *TUBB3*, as it has at least 16 annotated transcripts and one pseudogene. Per the HUGO Gene Nomenclature Committee (HGNC) page, the approved and preferred identifiers for *TUBB3* are NCBI (National Center for Biotechnology Information) Gene ID 10381; HGNC 20772; MANE Select: NM_006086 (RefSeq transcript) & ENST00000315491.12 (ENST identifier); and Ensemble: ENSG00000258947. Below, we use the *TUBB3* RefSeq transcript NM_006086, resulting in the 450 amino acid protein. Some papers and clinical labs, however, have used NM_001197181, which results in a 378 aa protein referred to as tubulin beta-3 chain isoform (TUBB3 tubulin beta 3 class III [*Homo sapiens* (human)] – Gene – NCBI). The use of this alternate transcript is pervasive enough that for many, but not all, entries in the NCBI ClinVar database^1^ both protein changes are displayed. Additionally, there is currently a duplicate entry for TUBB3 in the gnomAD resource^2^ and a search for TUBB3 currently defaults to an incorrect Ensembl gene ID ENSG00000198211.8 and Ensembl canonical transcript ENST00000556922.1, which are associated with a novel gene and 797 amino acid protein named MC1R-TUBB3 readthrough. This can be bypassed by selecting the second option in the Gnomad drop down, ENSG00000258947, which maps to the correct MANE Select canonical TUBB3 transcript.

The approved and preferred KIF21A HGNC identifiers are NCBI Gene ID 55605; HGNC 19349; MANE Select: NM_001173464.2 (RefSeq transcript) & ENST00000361418.10 (ENST identifier); Ensemble: ENSG00000139116. Below, we use the 38-exon NM_01173464 transcript, the most highly conserved and expressed canonical transcript. This is the transcript used by Ensembl and in most publications. Some papers and clinical labs do, however, use NM_017641 and multiple entries for the same variants can be listed in ClinVar. Thus, care must be taken to determine that the same, and preferably the preferred, transcript is being referenced for variant nomenclature and comparisons.

Below, we review the phenotypes reported with specific *TUBB3* variants, as summarized in Figures 1–4 and Tables 1–3. Using the Broad “PER viewer” (Pérez-Palma et al., 2020) at https://per.broadinstitute.org/, we also list if variants resulting in the same amino acid substitution in other β-tubulin genes have been reported to cause neurodevelopmental disorders.

**Table 1:** TUBB3-CFEOM variant phenotypes.

NM_006086	Variant	R62Q	S78T	R262C	E421D	D417N	D417H	E410V	E410K	R262H	A302T	R380C
	Nucleotide	185G > A	232 T > A	784C > T	1263G > C	1249G > A	1249G > C	1229A > T	1228G > A	785G > A	904G > A^2^	1138C > T
Pedigree Information	# Pedigrees^1^	1	1	20	1	7	2	1	17	15^3^	4	6
	# M/F/U/fetus	1/1/0/0	1/1/0/0	50+/50+/0/0	3/0/0/0	12/7/1/0	2/4/0/0	1/0/0/0	12/8/0/0	10/5/1/0	2/5/0/0	3/1/2/1
	Inheritance	AD	AD	AD>DN	AD	AD/DN	AD	DN	AD<DN	DN	AD/DN/G	DN/G
	Eldest	Adult	29y	85y	Adult	Adult	70s	8y	Adult	25y	Adult	10y
Peripheral NS dysfunction	Kallmann	no	no	no	no	no	51–75%	yes	76–100%	76–100%	no	no
	CN II: ONH	no	no	no	no	no	no	no	1–25%	26–50%	no	no
	CN III: CFEOM	50%	100%	76–100%	100%	51–75%	100%	yes	100%	100%	76–100%	26–50%
	CN VII: FW	no	no	no	51–75%	no	76–100%	yes	100%	100%	no	no
	CN IX-XII: Sw.	no	no	no	no	no	no	no	no	51–75%	no	no
	CN X: VC	no	no	no	no	no	no	no	76–100%	51–75%	no	no
	PN	no	no	no	no	IP 12-45y	teens-60’s	NA	~20 y	1-10y	no	no
	C contractures	no	no	no	no	no	51–75%	no	no	76–100%	no	no
	Cyclic vomiting	no	no	no	no	no	no	no	26–50%	1–25%	no	no
	Cardiac arrhy	no	no	no	no	no	26–50%	no	1–25%	1–25%	no	no
Central NS dysfunction	Microcephaly	no	no	no	no	no	no	no	1–25%	26–50%	no	1–25%
	Strabismus	S NA	S NA	S NA	S NA	S NA	S NA	S NA	S NA	S NA	S NA	S NA
	Nystagmus	N NA	N NA	1–25%	N NA	1–25%	N NA	yes	1–25%	N NA	N NA	N NA
	OMA	nr	nr	nr	nr	nr	nr	nr	nr	nr	nr	nr
	Seizures	no	no	no	no	no	no	no	1–25%	1–25%	no	no
	Motor delay	no	no	no	no	no	26–50%	yes	76–100%	76–100%	no	1–25%
	LD	no	no	no	76–100%	1–25%	LD NA	no	LD NA	LD NA	26–50%	LD NA
	ID/Social	no	no	no	no	no	1–25%	yes	76–100%	76–100%	no	76–100%
Brain MRI abnormality	Cerebral cortex	no	no	no	26–50%	no	no	no	1–25%	1–25%	no	26–50%
	AC	no	nr	76–100%	1–25%	76–100%	26–50%	yes	76–100%	76–100%	76–100%	21–50%
	CC	no	no	1–25%	no	1–25%	51–75%	yes	76–100%	76–100%	76–100%	76–100%
	White matter	no	no	no	no	no	51–75%	no	76–100%	76–100%	no	no
	Basal ganglia	no	no	mild	no	1–25%	no	no	no	76–100%	no	76–100%
	Cerebellum	no	no	no	no	1–25%	no	no	no	76–100%	no	51–75%
	Brainstem	no	no	no	no	no	no	no	1–25%	76–100%	no	26–50%

^1^Corresponding references reporting each variant are provided in the main text. ^2^Present in GnomAD with AF of 3.99e-6. ^3^Pedigree with monozygotic twins. no, absent; nr, not reported or mentioned in publication(s); percentages, percentage range of affected individuals for whom the phenotype has been reported. # Pedigrees, number of pedigrees reported in the literature; # M/F/U/fetus, number of affected individuals who are male, female, unknown or were a fetal case. AD, autosomal dominant; DN, *de novo*; G, germline mosaic. Eldest, eldest reported individual to date harboring the variant. Y, years of age. Kallmann, anosmia and hypogonadotropic hypogonadism and/or hypoplasia/absence of olfactory sulci and bulbs. CN II: ONH, optic nerve: optic nerve hypoplasia. CN III: CFEOM, oculomotor nerve: congenital fibrosis of the extraocular muscles. CN VII: FW, facial nerve: congenital facial weakness. CN IX-XII: Sw., lower cranial nerves: swallowing dysfunction. CN X: VC, vagal nerve: vocal cord paralysis. PN, mixed sensorimotor axonal polyneuropathy. C contractures, congenital contractures. Cardiac Arrhy, sick sinus syndrome with or without a pacemaker. S NA, strabismus not relevant given CFEOM. N NA, nystagmus not relevant given CFEOM. OMA, oculomotor apraxia. LD, learning disorder. LD NA, LD not relevant given ID/Social disabilities. ID/Social, intellectual and social disabilities (including ASD). Cerebral cortex, malformation of cortical development. AC, hypoplasia or absence of anterior commissure. CC, hypoplasia or absence of corpus callosum. White matter, paucity of central white matter and/or aberrant fascicles or signal where noted. Basal ganglia, presence of any abnormality ranging from mild asymmetry of caudate to full malformation. Cerebellum, malformation of vermis and/or hemispheres. Brainstem, hypoplasia or malformation.

**Table 2:** TUBB3-MCD variant phenotypes.

NM_006086	Variant	R46G	G82R	T178M	E205K	S230L	P243L	L273F	E288K	A302V	T312M	M323V	P357L	M388T	M388V
	Nucleotide	136 C > G	244 G > A	533 C > T^2^	613 G > A	689 C > T	728 C > T	817 C > T	862 G > A	905 C > T	935 C > T	967 A > G	1070 C > T	1163 T > C	1162 A > G
Pedigree Information	# Pedigrees^1^	1	1	4	2	3	2	1	7	1	1	2	3	1	1
	# M/F/U/fetus	0/1/0/0	1/0/0/0	0/2/1/1	3/2/0/0	1/1/1/0	1/1/0/1	0/0/0/1	4/1/1/1	2/3/0/0	1/2/0/1	3/0/0/0	2/1/0/0	1/0/0/0	0/0/0/1
	Inheritance	DN	DN	DN/ND	AD/DN	DN/ND	AD	DN	AD<DN	AD^3^	AD	AD/DN	DN	ND	DN
	Eldest	2y	5y	7y	14y	34y	31y	fetus	4yo	25y	40y	38y	6.5y	0.3y	fetus
Peripheral NS dysfunction	Kallmann	nr	nr	nr	nr	nr	nr	nr	nr	nr	nr	nr	nr	nr	yes
	CN II	nr	nr	nr	nr	nr	nr	nr	nr	nr	nr	nr	nr	yes	yes
	CN III: CFEOM	nr	no	no	no	nr	nr	nr	nr	no	nr	no	nr	nr	no
	CN VII	nr	nr	nr	nr	nr	nr	nr	nr	nr	nr	nr	nr	nr	nr
	CN IX, X	nr	nr	nr	nr	nr	nr	nr	nr	nr	nr	nr	nr	nr	nr
	CN X	nr	nr	nr	nr	nr	nr	nr	nr	nr	nr	nr	nr	nr	nr
	PN	nr	nr	nr	nr	nr	nr	nr	nr	nr	nr	nr	nr	nr	nr
	C contractures	nr	nr	nr	nr	nr	nr	nr	nr	nr	nr	nr	nr	nr	nr
	Cyclic vomiting	nr	nr	nr	nr	nr	nr	nr	nr	nr	nr	nr	nr	nr	nr
	Cardiac aryth	nr	nr	nr	nr	nr	nr	nr	nr	nr	nr	nr	nr	nr	nr
Central NS dysfunction	Microcephaly	yes	no	26–50%	no	26–50%	26–50%	no	no	no	1–25%	no	no	yes	yes
	Strabismus	nr	yes	26–50%	26–50%	nr	26–50%^4^	nr	26–50%	100%	nr	51–75%	51–75%	nr	nr
	Nystagmus	nr	nr	1–25%	nr	nr	nr	nr	1–25%	26–50%	nr	100%	nr	nr	nr
	OMA	nr	nr	nr	nr	nr	nr	nr	26–50%	nr	nr	nr	51–75%	nr	nr
	Seizures	nr	no	51–75%	26–50%	no	EEG only	nr	1–25%	no	nr	no	26–50%	yes	nr
	Motor delay	nr	yes	100%	100%	100%	yes	nr	76–100%	26–50%	26–50%	51–75%	100%	yes	nr
	LD	nr	nr	nr	NA	nr	no	nr	NA	nr	26–50%	NA	nr	nr	nr
	ID/Social	nr	yes	100%/ND	100%	100%	no	nr	76–100%	mild	no	100%	51–75%	yes	nr
Brain MRI abnormality	Cerebral cortex	yes	yes	51–75%	100%	26–50%	100%	yes	51–75%	100%	51–75%	100%	100%	yes	yes
	AC	nr	nr	nr	nr	nr	nr	nr	nr	nr	nr	nr	nr	nr	nr
	CC	yes	yes	100%	100%	100%	yes	yes	76–100%	26–50%	100%	100%	100%	nr	yes
	White matter^5^	nr	nr	26–50%	nr	100%	nr	no	nr	nr	nr	nr	26–50%	nr	yes
	Basal ganglia	nr	yes	100%	100%	100%	nr	yes^5^	76–100%	26–50%	no	100%	100%	nr	yes
	Cerebellum	yes	yes	100%	100%	no	nr	yes	100%	100%	100%	100%	51–75%	yes	yes
	Brainstem	nr	yes	26–50%	100%	no	nr	yes	100%	26–50%	no	51–75%	100%	nr	yes

^1^Corresponding references reporting each variant are provided in the main text. ^2^Present in GnomAD with AF of 3.98e-6. ^3^One pedigree with heterozygous mother and 3 offspring, while a fourth offspring harbors homozygous variant. ^4^One affected individual reported with “uncorrectable strabismus from birth” which could be interpreted as CFEOM. ^5^Abnormal ganglionic eminence. no, absent; nr, not reported or mentioned in publication(s); percentages, percentage range of affected individuals for whom the phenotype has been reported. # Pedigrees, number of pedigrees reported in the literature; #M/F/U/fetus, number of affected individuals who are male, female, unknown or were a fetal case. AD, autosomal dominant; DN, *de novo*; ND, not determined. Eldest, eldest reported individual to date harboring the variant. Y, years of age. Kallmann, anosmia and hypogonadotropic hypogonadism and/or hypoplasia/absence of olfactory sulci and bulbs. CN II: ONH, optic nerve: optic nerve hypoplasia. CN III: CFEOM, oculomotor nerve: congenital fibrosis of the extraocular muscles. CN VII: FW, facial nerve: congenital facial weakness. CN IX-XII: Sw., lower cranial nerves: swallowing dysfunction. CN X: VC, vagal nerve: vocal cord paralysis. PN, mixed sensorimotor axonal polyneuropathy. C contractures, congenital contractures. Cardiac Arrhy, sick sinus syndrome with or without a pacemaker. S NA, strabismus not relevant given CFEOM. N NA, nystagmus not relevant given CFEOM. OMA, oculomotor apraxia. LD, learning disorder. LD NA, LD not relevant given ID/Social disabilities. ID/Social, intellectual and social disabilities (including ASD). Cerebral cortex, malformation of cortical development. AC, hypoplasia or absence of anterior commissure. CC, hypoplasia or absence of corpus callosum. White matter, paucity of central white matter and/or aberrant fascicles or signal where noted. Basal ganglia, presence of any abnormality ranging from mild asymmetry of caudate to full malformation. Cerebellum, malformation of vermis and/or hemispheres. Brainstem, hypoplasia or malformation.

**Table 3:** TUBB3-CFEOM/MCD and TUBB3-nonCFEOM/nonMCD variant phenotypes.

		TUBB3-CFEOM/MCD		TUBB3-nonCFEOM/nonMCD					
NM_006086	Variant	G71R	G98S	R46W	V175L	T238A	V255I	Q292E	R391L
	Nucleotide	211G > A	292G > A	136C > T	523G > C	712A > G	763G > A	874C > G	1172G > T
Pedigree Information	# Pedigrees^1^	3	4	2	1	1	2	1	2
	# M/F/U/fetus	1/2/0/0	1/1/2/0	2/0/0/0	0/1/0/0	0/1/0/0	1/1/0/0	1/0/0/0	1/1/0/0
	Inheritance	DN	DN	DN/ND	DN	DN	DN	DN	DN
	Eldest	23y	2y	11y	1 m	7yo	16y	8 m	14y
Peripheral NS dysfunction	Kallmann	51–75%^2^	26–50%^2^	nr	nr	nr	nr	nr	nr
	CN II: ONH	no	26–50%	nr	yes	no	26–50%	nr	nr
	CN III: CFEOM	100%	51–75%	nr	nr	no	no	nr	no
	CN VII: FW	no	no	nr	nr	nr	nr	nr	no
	CN IX-XII: Sw.	no	no	nr	nr	nr	nr	nr	nr
	CN X: VC	no	no	nr	nr	nr	nr	nr	nr
	PN	no	no	nr	nr	nr	nr	nr	nr
	C contractures	no	no	nr	nr	nr	nr	nr	nr
	Cyclic vomiting	no	no	nr	nr	nr	nr	nr	nr
	Cardiac arrhy	no	no	nr	nr	nr	nr	nr	nr
Central NS dysfunction	Microcephaly	no	26–50%	26–50%	no	no	no	no	100%
	Strabismus	S NA	26–50%	26–50%^3^	no	no	26–50%	no	no
	Nystagmus	26–50%	26–50%	nr	nr	no	nr	nr	nr
	OMA	nr	nr	nr	nr	nr	nr	nr	nr
	Seizures	no	no	26–50%	EEG abnl	no	26–50%	no	100%
	Motor delay	100%	100%	100%	NA	yes	100%	yes	100%
	LD	LD NA	LD NA	LD NA	NA	LD NA	LD NA	nr	LD NA
	ID/Social	100%	100%	100%	NA	yes	100%	nr	100%
Brain MRI abnormality	Cerebral cortex	100%	100%	no	WWS-like	no	no	no	no
	AC	100%	26–50%	nr	nr	nr	nr	nr	nr
	CC	100%	100%	26–50%	yes	yes	100%	yes	100%
	White matter	51–75%	26–50%	nr	nr	nr	yes	nr	nr
	Basal ganglia	100%	100%	26–50%	nr	yes	yes	nr	nr
	Cerebellum	100%	100%	nr	yes	yes	100%	nr	nr
	Brainstem	100%	100%	nr	yes	yes	no	nr	nr

^1^Corresponding references reporting each variant are provided in the main text. ^2^Refers to olfactory system anomalies. ^3^One affected individual reported with “divergent squint, vertical strabismus” which could be interpreted as CFEOM. no, absent; nr, not reported or mentioned in publication(s); percentages = percentage range of affected individuals for whom the phenotype has been reported. # Pedigrees, number of pedigrees reported in the literature; #M/F/U/fetus, number of affected individuals who are male, female, unknown or were a fetal case. AD, autosomal dominant; DN, *de novo*; ND, not determined. Eldest, eldest reported individual to date harboring the variant. Y, years of age. Kallmann, anosmia and hypogonadotropic hypogonadism and/or hypoplasia/absence of olfactory sulci and bulbs. CN II: ONH, optic nerve: optic nerve hypoplasia. CN III: CFEOM, oculomotor nerve: congenital fibrosis of the extraocular muscles. CN VII: FW, facial nerve: congenital facial weakness. CN IX-XII: Sw., lower cranial nerves: swallowing dysfunction. CN X: VC, vagal nerve: vocal cord paralysis. PN, mixed sensorimotor axonal polyneuropathy. C contractures, congenital contractures. Cardiac Arrhy, sick sinus syndrome with or without a pacemaker. S NA, strabismus not relevant given CFEOM. N NA, nystagmus not relevant given CFEOM. OMA, oculomotor apraxia. LD, learning disorder. LD NA, LD not relevant given ID/Social disabilities. ID/Social, intellectual and social disabilities (including ASD). Cerebral cortex, malformation of cortical development. AC, hypoplasia or absence of anterior commissure. CC, hypoplasia or absence of corpus callosum. White matter, paucity of central white matter and/or aberrant fascicles or signal where noted. Basal ganglia, presence of any abnormality ranging from mild asymmetry of caudate to full malformation. Cerebellum, malformation of vermis and/or hemispheres. Brainstem, hypoplasia or malformation.

### TUBB3-CFEOM syndromes

A subset of heterozygous *TUBB3* missense variants cause CFEOM (Table 1). While some result in isolated CFEOM, most are accompanied by additional cranial/peripheral nerve and central white matter maldevelopment. Clinically, these can include anosmia (CN I) with hypogonadotropic hypogonadism and failure to go through puberty (Kallmann syndrome), optic nerve hypoplasia (CN II), facial weakness (CN VII), swallowing dysfunction (CN IX-XII), vocal cord paralysis (CN X), progressive axonal motor-sensory polyneuropathy, congenital joint contractures, cyclic vomiting, cardiac arrhythmias, and intellectual and social disabilities. For isolated CFEOM, the MRI is as described in the introduction and below. For syndromic cases, MRI can reveal hypoplasia or absence of additional CNs, thinning or absence of the anterior commissure and corpus callosum, thinning and/or mistargeting of additional central white matter tracts, malformed basal ganglia, and cerebellar vermis (Figures 1, 2; Tischfield et al., 2010; Chew et al., 2013; Grant et al., 2018; Whitman et al., 2021). With only a few exceptions mentioned below, most subjects do not have MCD.

Basal ganglia (BG) malformations are a hallmark of most TUBB3-CFEOM and TUBB3-MCD variants as well as many other tubulinopathies, and include varying degrees of abnormal rotation and asymmetrical enlargement of the caudate head and hypoplasia of the caudate body, with accompanying dilatation of the anterior horn of the lateral ventricle, hypoplasia of the anterior limb of the internal capsule with fusion of the caudate head and underlying putamen, and rounded globular-appearing thalami (Figures 2, 3).

Correlations between the specific TUBB3 amino acid substitution and the resulting phenotype were observed in the first report of human TUBB3-CFEOM variants (Tischfield et al., 2010). These have held up to a great degree, permitting the definition of a series of TUBB3 syndromes referred to by the amino acid substitution. Below, the TUBB3-CFEOM syndromes are divided into those with multiple or a single proband reported and summarized generally in order of increasing phenotypic severity.

#### TUBB3-CFEOM variants without MCD and with multiple probands reported

**TUBB3 R262C syndrome [784C > T]**: Over 100 individuals from 20 pedigrees have been reported; 14 AD inheritance, 6 *do novo* Tischfield et al., 2010; Ceylan et al., 2017; Stark et al., 2017; Dillon et al., 2018; Jia et al., 2022). Affected individuals were born with isolated CFEOM that could be asymmetrical and vary in severity within a pedigree. Most commonly individuals had ptosis, infraducted eyes, absent upgaze, variably restricted down and horizontal gaze (Figure 1). On occasion, the variant was not penetrant or resulted in isolated restriction of upgaze with no ptosis (Doherty et al., 1999; Tischfield et al., 2010). MRI revealed thin or absent CN III and, in some cases, thin or absent CN VI. The superior rectus and levator palpebrae superioris (LPS) EOMs were hypoplastic and, in some cases, the medial, inferior, and lateral rectus EOMs were hypoplastic as well (Demer et al., 2010). Affected individuals also had thin or absent anterior commissure and occasionally slight thinning of the corpus callosum posterior body with corpus callosal agenesis (CCA) reported only once. The head of the caudate nucleus could appear asymmetrical (Figure 2).

**TUBB3 A302T syndrome [904G > A]**: Seven individuals across 4 pedigrees have been reported; one *de novo* singleton, two AD pedigrees, two siblings with germline mosaicism (Tischfield et al., 2010; Jang et al., 2022). CFEOM varied from mild unilateral elevation deficiency without ptosis to bilateral, severe CFEOM. While 5 of the subjects did not have developmental concerns, the siblings with germline mosaicism had learning disabilities. MRI revealed CFEOM changes and normal to diffuse corpus callosum hypoplasia (Figures 1, 2). As noted below, A302V causes TUBB3-MCD.

**TUBB3 D417N syndrome [1249G > A]**: Twenty individuals across 7 pedigrees have been reported; 5 familial, 2 *de novo* (Tischfield et al., 2010; Zhang and Xing, 2013; Hong et al., 2015; Sainio et al., 2022). Subjects had CFEOM and/or onset of a peripheral neuropathy in the second or third decade of life (Figure 1). Affected members of one family included those with both CFEOM and PN, only PN, or only CFEOM; in another the affected members over 3 generations had isolated CFEOM with no PN mentioned; in a third the affected members had onset of peripheral neuropathy between 12 and 45 years of age in the absence of CFEOM. Most lacked additional non-neuropathy motor symptoms or cognitive impairments, although one family included several boys with learning disabilities and developmental delays. MRI revealed CFEOM changes and relatively minor and variable anterior commissure and corpus callosum hypoplasia, with or without basal ganglia malformations (Figure 2). D417N has also been reported in TUBB2B (causing polymicrogyria) and TUBB8 (causing oocyte maturation defect).

**TUBB3 R380C syndrome [1138C > T]**: Seven individuals from six pedigrees have been reported; five singletons (*de novo* when tested) including a 26 week fetus, and two siblings with evidence of germline mosaicism (Pieh et al., 2003; Tischfield et al., 2010; Srivastava et al., 2014; Romaniello et al., 2017; Goergen et al., 2021; Klee et al., 2021; Jia et al., 2022). The sibs and one simplex case had CFEOM (Figure 1), while 2 subjects lacked ptosis and for two others the eye movement abnormalities and ocular details were not provided. Unlike other neurologically syndromic TUBB3-CFEOM variants, facial palsy, contractures, and neuropathy have not been documented.

Neuroimaging was reported for six subjects (Figure 2), including the fetus. CN III was hypoplastic to absent in those with CFEOM. When noted, the corpus callosum was abnormal and there was corpus callosal agenesis in the two sibs. All had basal ganglia malformations and brainstem hypoplasia. The cerebellar vermis was mildly hypoplastic in at least four subjects with cerebellar hemisphere malrotation in one. Notably, the fetus had severe ventriculomegaly L > R, abnormal opercularization, and dysgyria, while MCD was not reported in the living subjects. R380C has also been reported in TUBB2B (causing perisylvian polymicrogyria).

**TUBB3 D417H syndrome [1249G > C]**: Six individuals across 2 AD pedigrees have been reported (Tischfield et al., 2010; Van Ryzin et al., 2016). Subjects have CFEOM, peripheral neuropathy with variability in severity and age of onset (a mother became symptomatic in her 7th decade while her daughter was symptomatic at a young age), Kallmann syndrome, congenital facial weakness, congenital wrist/finger joint contractures, and learning or intellectual disabilities. One individual died of cardiac-related causes in her early 40’s and two others have been evaluated for sick sinus syndrome. MRI of two individuals revealed thin corpus callosum, frontal lobe interdigitation, dysmorphic olfactory sulci and bulbs (consistent with clinical Kallmann syndrome) (Figure 2), and small or absent oculomotor and facial nerves.

**TUBB3 E410K syndrome [1228G > A]**: Seventeen individuals from 14 pedigrees have been reported; thirteen cases were *de novo*, while one affected mother who harbored a *de novo* variant transmitted it to her three affected sons (Tischfield et al., 2010; Chew et al., 2013; Balasubramanian et al., 2015; Patel et al., 2017; Romaniello et al., 2017; Grant et al., 2018; Nakamura et al., 2018; Dentici et al., 2020; Dong et al., 2020). E410K results in a stereotypical syndromic presentation. All individuals had severe CFEOM with both eyes fixed in a hypotropic and exotropic (down and out) position (Figure 1). All had congenital anosmia, facial weakness, and facial dysmorphisms. Fetal distress, however, occurred in a minority of newborns and tracheostomy was rarely required, with only a few diagnosed with vocal cord paralysis. Stature and head circumference were under 50% while 4 of 17 had microcephaly. All older individuals, except the mother who transmitted her variant, had Kallmann syndrome. Most developed cyclic vomiting with age of onset between 3 and 20 years of age. All older individuals developed a peripheral neuropathy with symptom onset in the teens although electrophysiological studies detected neuropathy at a younger age, and only one reported joint contractures with unknown age of onset. Most, but not all, with formal testing had intellectual disabilities, and some had ASD. Importantly, one E410K subject had sinus arrhythmia during vomiting episodes, another had cough syncope, and a third had sinus node dysfunction requiring pacemaker placement.

MRIs revealed hypoplasia/aplasia of CN III and CN VII, small EOMs, olfactory bulb and sulcus dysgenesis, paucity of cerebral white matter, thin to absent anterior commissure, corpus callosum that ranged from posterior hypoplasia to complete agenesis, and normal basal ganglia and cerebellum. Two individuals had abnormal brainstem morphology, and one case was reported to have MCD (simplified gyral pattern) for which MRI were not provided. Notably, basal ganglia and thalami appeared normal (Figures 1, 2).

Diffusion tensor imaging (DTI) was reported for seven individuals (Grant et al., 2018). Subjects had decreased corpus callosum volume, an aberrant corticospinal tract trajectory, and bilateral absence of the dorsal language network long segment. In addition, the pediatric subjects had diffuse decreases in fractional anisotropy while adult subjects had white matter volume loss associated with decreased cortical surface area; these age-related changes were felt to result from microscopic intrahemispheric misguided axons that were pruned during maturation. These findings suggested that TUBB3 E410K causes defects in both peripheral central axon growth, guidance, and maintenance. Of note, the E410K substitution has also been reported in the TUBB4A isotype (causing torsion dystonia, hypomyelinating leukodystrophy, spastic paraparesis) and in the TUBB8 isotype (causing male infertility).

**TUBB3 R262H syndrome [785G > A]**: Sixteen individuals from fifteen pedigrees have been reported; variants were *de novo* in 13 pedigrees including monozygotic twins, and not tested in two simplex cases (Tischfield et al., 2010; Whitman et al., 2021; Frisk et al., 2022; Jia et al., 2022). This variant results in a strikingly syndromic presentation and is the most severe of the TUBB3-CFEOM syndromes identified to date (Figure 1). It encompasses all the E410K findings with the following additions: a higher frequency of microcephaly (50%); a higher frequency of vocal cord dysfunction with respiratory distress at birth frequently requiring tracheostomy; an earlier onset of peripheral neuropathy most typically in the first decade of life; congenital joint contractures; limited independent ambulation. Interestingly, all individuals old enough to show a hand preference were left-handed or, in one case, had poor use of both hands. Many young children had sinus tachycardia, though most reportedly did not require continued follow-up. Sinus node dysfunction requiring pacemaker placement has been reported in one adult.

MRI revealed changes similar to the TUBB3 E410K syndrome with the exception that basal ganglia malformations were present with TUBB3 R262H but not E410K (Figure 2). In addition, although cortical malformations were not identified in the original MRI report (Tischfield et al., 2010), three recently reported subjects had Sylvian fissure asymmetries, one of whom also had polymicrogyria (Whitman et al., 2021), and a fourth subject had cortical dysgenesis (Jia et al., 2022). Thus, the incidence of MCD in this syndrome remains unclear. The R262H substitution has also been reported in TUBB2A (causing arthrogryposis multiplex congenita) and TUBB4A (causing hypomyelinating leukodystrophy).

#### TUBB3-CFEOM variants without MCD and with a single proband reported

**TUBB3 R62Q [185G > A]**: One pedigree has been reported (Tischfield et al., 2010). A son had isolated CFEOM and normal MRI imaging, and inherited the variant from his phenotypically unaffected mother (Figure 2).

**TUBB3 S78T [232 T > A]**: One AD pedigree with 2 affected individuals has been reported (Jia et al., 2022). The mother had isolated bilateral CFEOM, and the child had isolated unilateral CFEOM and nystagmus.

**TUBB3 E410V [1229A > T]**: One 8-year-old male with a *de novo* variant has been reported (MacKinnon et al., 2014; Heidary and Hunter, 2019). His presentation was not distinguishable from E410K subjects; he had ASD, was working at grade level, and had not developed cyclic vomiting or PN.

**TUBB3 E421D [1263G > C]**: One AD, syndromic CFEOM pedigree of a father and two affected sons has been reported (Thomas et al., 2019). The father had bilateral ptosis and upgaze limitation, while both sons lack ptosis and had unilateral vertical gaze restriction, referenced as monocular elevation deficiency. All three had mild learning difficulties and mild ataxia, and two had mild facial weakness. The father had decreased sensation and depressed deep tendon reflexes with normal nerve conductions. MRI of the sons revealed small anterior commissure with normal corpus callosum, olfactory system and basal ganglia. One son had a focal cortical dysplasia in the left insular cortex; while this could be considered a CFEOM/MCD combined variant, focal cortical dysplasias are a class of localized regions of malformed cortex that are considered separate from more widespread MCDs (Blümcke et al., 2011).

Of note, a second substitution at this same residue in TUBB2B, TUBB2B E421K, was reported in a dominant family with CFEOM and polymicrogyria and, in yeast, the variant stabilized microtubules and reduced kinesin localization on microtubules (Cederquist et al., 2012).

### TUBB3-MCD syndromes

While many individuals with TUBB3-MCD variants have strabismus and some have nystagmus, these variants are not associated with CFEOM or with known dysfunction of other CNs. Moreover, while there may be some phenotype–genotype correlations, these are far less evident than with the TUBB-CFEOM variants, and thus these variants are listed in N- to C-terminal amino acid order are not referred to as distinct amino acid-based syndromes (Table 2 and Figures 3, 4).

#### TUBB3-MCD variants without CFEOM and with multiple probands reported

**TUBB3 T178M [533C > T]**: Three live-born subjects and one fetus, at least 3 *de novo*, have been reported (Poirier et al., 2010; Bahi-Buisson et al., 2014; Boissel et al., 2018; Strauss et al., 2018; Park et al., 2021). At 20 weeks gestational age, ultrasound of the fetus revealed a small cerebellum, tri-ventriculomegaly, and fenestration of the septum. Fetopsy revealed ventriculomegaly, corpus callosal agenesis, heterotopic white matter fascicles, severely hypoplastic corticospinal tract, and cerebellar hypoplasia. Cortical anomalies were not described, and the age of the fetus was not provided. Of the three live-born children, a five-year-old girl was described as microcephalic, with axial hypotonia, spastic tetraplegia, intermittent strabismus, multidirectional nystagmus, and refractory neonatal epilepsy. On imaging she had corpus callosal agenesis, cortical gyral disorganization later described as generalized polymicrogyria, basal ganglia malformations, hypoplastic vermis with severely dysplastic cerebellar hemispheres, and hypoplastic brainstem. A second live-born female was diagnosed with semilobar holoprosencephaly on routine prenatal US and had refractory neonatal epilepsy and intermittent strabismus. At 6 years of age she had daily seizures, was non-verbal and non-ambulatory, and required gastrostomy tube feeding for swallowing dysfunction and oral aversion. On postnatal MRI she had a thin corpus callosum with reduced white matter, cortical dysgyria, malformed basal ganglia, small and disorganized cerebellar vermis, and small and asymmetric brainstem. The third live-born infant was simply described as having cortical dysplasia. Figure 3 presents MR imaging of a fifth individual harboring this variant. T178M has also been reported in TUBB2A (causing nervous system abnormality) and TUBB4A (causing hypomyelinating leukodystrophy).

**TUBB3 E205K [613G > A]**: Five subjects from 2 pedigrees have been reported; a boy harboring a *de novo* variant and an affected mother with three affected offspring (Poirier et al., 2010; Bahi-Buisson et al., 2014). Details were provided for the singleton and two of the siblings at 8, 10, and 14 years of age. The singleton had borderline microcephaly, moderate intellectual disability, strabismus, and mild spasticity, while the siblings both had severe intellectual disability and axial hypotonia and one had prolonged febrile seizures. MRI revealed multifocal polymicrogyria, thin or thick corpus callosum, malformed basal ganglia, dysplastic cerebellar vermis, and hypoplastic brainstem in all three children. E205K has also been reported in TUBB8 (causing oocyte maturation arrest).

**TUBB3 S230L [689C > T]**: Three unrelated subjects (at least one *de novo*) have been reported (Shimojima et al., 2016; Romaniello et al., 2019; Kars and Ozcelik, 2021). One child had a small head size at birth (−2.6SD) and at 16 months (−2.4SD). She rolled over at 8 months and, at 16 months, she was able to sit and communicated with gestures. MRI revealed corpus callosum thinning and a left frontal cortical malformation. A 34-year-old man was reported with this variant who was part of a tubulinopathy epilepsy case series and had no history of seizures and, by MRI, had no MCD; no further clinical details were provided. No data were provided for the third individual.

**TUBB3 P243L [728C > T]**: Three subjects from 2 pedigrees have been reported; a carrier mother with pregnancy termination, and a 12-year-old boy with undetermined inheritance (Reches et al., 2018; Romaniello et al., 2019; Blumkin et al., 2020; Yaron et al., 2022). The mother had right-sided weakness for which she received physical therapy as a child, and ‘uncorrectable’ congenital strabismus. She recalled childhood neuroimaging as ‘abnormal.’ Ultrasound of her fetus at 26 weeks gestational age revealed abnormal cerebral sulcation and suspected cortical migration defect. Her fetus’ MRI revealed an asymmetrical complex brain malformation most consistent with polymicrogyria or hemimegalencephaly. Fetopsy revealed pachygyria, markedly variable cortical thickness, abnormal Sylvian fissures, small malformed temporal lobes, short corpus callosum, and hypoplastic inferior cerebellar vermis with normal appearing basal ganglia. The 12-year-old boy was reported as part of an epilepsy series and had abnormal EEG, no seizures, and MRI with perisylvian polymicrogyria with a thin and short corpus callosum, basal ganglia malformations, and brainstem asymmetry.

**TUBB3 E288K [862G > A]**: One fetus and 6 live born subjects (at least 6 *de novo*) have been reported (Oegema et al., 2015; Fukumura et al., 2016; Romaniello et al., 2017; Boissel et al., 2018; Accogli et al., 2020; Lee et al., 2020). Fetopsy at ~27 weeks gestational age revealed triventriculomegaly, simplified gyration of the frontal and temporal lobes, moderate pontocerebellar hypoplasia, and abnormal corticospinal tracts. The live-born subjects, assessed between 2 and 4 years of age, all had developmental delay, while one had seizures and two others had abnormal EEGs. Three had hypotonia. Three had strabismus, two had ocular motor apraxia, and one had nystagmus. One had unilateral autonomic dysfunction with hypohidrosis but normal cardiovascular function and no periphreal neuropathy. By MRI, the cortex was described as dysgyria, bilateral dysgyria with bilateral insular subcortical nodular heterotopia, irregular gyral pattern, and isolated lissencephaly with ventriculomegaly in 4 subjects, and no MCD was detected in two. The corpus callosum was hypoplastic or dysmorphic and basal ganglia malformed in all live-born subjects, and all but one had cerebellar and brainstem malformations. E288K has also been reported in TUBB2B (causing pachygyria).

**TUBB3 M323V [967A > G]**: Three subjects have been reported; a father and son and one infant harboring a *de novo* variant (Poirier et al., 2010; Bahi-Buisson et al., 2014; Jin et al., 2021). All three had learning or intellectual disabilities and nystagmus, and the father and son also had strabismus and hypotonia. MRI revealed gyral disorganization, hypoplastic or asymmetric corpus callosum, basal ganglia malformations, and vermian dysplasia in all, dysplastic cerebellar hemispheres in the father, and brainstem hypoplasia in the father and son.

**TUBB3 P357L [1070C > T]**: Three unrelated subjects have been reported, all *de novo* (Oegema et al., 2015; Cabet et al., 2021). All had motor delays, one had an IQ at “low end of normal” and one had seizures. Two were diagnosed with ocular motor apraxia and two had strabismus. MRI revealed MCDs; two had diffusely irregular gyration and sulcation and the third had frontal asymmetry and lack of sylvian fissure operculization. Two had abnormal cerebellar vermes and all had corpus callosum hypoplasia, dysmorphic basal ganglia and asymmetric brainstem.

#### TUBB3-MCD variants without CFEOM and with a single proband reported

**TUBB3 R46G [136C > G]**: A *de novo* variant in a microcephalic 2-year-old girl with intellectual disability and motor delays. MRI revealed multifocal polymicrogyria, dysmorphic and thin corpus callosum, and vermian dysplasia (Bahi-Buisson et al., 2014).

**TUBB3 G82R [244G > A]**: A *de novo* variant in a 5-year-old boy with axial hypotonia, moderate intellectual disability and strabismus. MRI revealed perisylvian and frontal multifocal polymicrogyria and microgyria, posterior corpus callosal agenesis, basal ganglia malformations, dysplasia of vermis but not cerebellar hemispheres, and brainstem hypoplasia (Poirier et al., 2010; Bahi-Buisson et al., 2014).

**TUBB3 L273F [817C > T]**: A *de novo* variant in a normocephalic male fetus at 23 weeks gestational age. Brain weight was low, and there was microlissencephaly with corpus callosal agenesis, basal ganglia abnormalities, and cerebellar vermis and brainstem hypoplasia (Alby et al., 2016).

**TUBB3 A302V [905C > T]**: Five members of one consanguineous pedigree have been reported (Poirier et al., 2010; Bahi-Buisson et al., 2014). The mother and three children were heterozygous and the youngest child homozygous for the variant. Clinical and imaging data were provided for only the mother and homozygous daughter. At 40 years of age, the mother was normocephalic and had mild intellectual disability, while her three-year-old daughter was reportedly severely affected, with borderline microcephaly (3rd percentile). Both had intermittent strabismus, and the daughter had multidirectional nystagmus and axial hypotonia. By MRI, both had a simplified frontoparietal gyral pattern, and the daughter also had poorly folded gyri, generalized undersulcation, and focal areas of thickened cortex. Both had thin corpus callosum and cerebellar vermian dysplasia. The daughter had malformed basal ganglia and a hypoplastic brainstem.

A302V is the only homozygous *TUBB3* variant in the literature, and phenotypic descriptions of the child as she ages and of her heterozygous siblings would be informative to better appreciate how the homozygous and heterozygous phenotypes compare. Finally, note that A302T causes TUBB3-CFEOM, suggesting that different substitutions at this residue can result in MCD vs. CFEOM phenotypes.

**TUBB3 T312M [935C > T]**: One pedigree with three living members (mother, son, daughter) and a terminated fetus have been reported (Blumkin et al., 2020; Nissenkorn et al., 2020). The family was identified during the mother’s second pregnancy when a routine 24-week gestational age ultrasound and follow-up MRI identified ventricular asymmetry, irregular frontal sulci, asymmetric Sylvian fissures and olfactory sulci, and vermian hypoplasia. Fetopsy at ~34 weeks gestational age revealed focal cortical thickening and unusually oriented frontal sulci and gyri and periventricular pseudocysts, thin corpus callosum, and vermian hypoplasia. Brain size, basal ganglia, cerebellar hemispheres and brainstem were unremarkable. Subsequent to identification of the *TUBB3* variant in the fetus, it was identified in the mother, her first-born son, and her then-current pregnancy. The mother was a late walker and her son had delayed speech and language skills, but their development, as well as that of the subsequent daughter, evaluated at 6 months of age, was otherwise normal. They had small head circumferences, the mother had mild tandem gait difficulties, and she and her son had mirror movements, while the daughter’s exam was normal. On MRI, the mother had a normal-appearing cortex while the children had dysgyria and/or sylvian fissure asymmetry. All had thin corpus callosum, normal basal ganglia, and cerebellar vermian hypoplasia with or without cerebellar hemisphere dysplasia. The mother had an asymmetric brainstem. Periventricular pseudocysts were identified on fetal imaging for both the terminated pregnancy and the daughter.

**TUBB3 M388V [1162A > G]**: *De novo* in a male fetus terminated at 27 weeks gestational age, reported multiple times (Poirier et al., 2010; Bahi-Buisson et al., 2014; Fallet-Bianco et al., 2014; Alby et al., 2016). Fetopsy revealed microcephaly, microlissencephaly, corpus callosal agenesis, olfactory bulb agenesis and optic nerve hypoplasia, basal ganglia malformations, cerebellar vermis and brainstem hypoplasia, absent corticospinal tracts, retrognathia, and camptodactyly. Immunostaining revealed abnormal cortical cytoarchitecture and neuronal differentiation in the neocortex, with aberrant fiber bundles in the superficial cortical layer and periventricular regions. M388V has also been reported in TUBB4A (causing hypomyelination with atrophy of the basal ganglia and cerebellum).

**TUBB3 M388T [1163 T > C]**: One male who died at 4 months of age has been reported; variant inheritance was not tested. Infant had severe congenital microcephaly (−4.1SD), optic atrophy, overall growth retardation, uncontrolled seizures, and developed no milestones. MRI revealed dysgyria with hypoplastic cerebellar vermis and hemispheres (Donato et al., 2018). M388T has also been reported in TUBB4A (causing hypomyelination with atrophy of the basal ganglia and cerebellum).

### TUBB3-CFEOM/MCD syndromes

Following the description of TUBB3-CFEOM and TUBB3-MCD variants, TUBB3 G71R and G98S substitutions were reported to cause both CFEOM and MCD as summarized below. In addition, mild MCDs have been reported with rare cases of R262H (Whitman et al., 2021), E410K (Patel et al., 2017) and R380C (Goergen et al., 2021), blurring the distinction between these two phenotypic categories. It remains to be determined if additional TUBB3-CFEOM variants result in MCDs that are simply below the resolution of current MRI technologies.

**TUBB3 G71R Syndrome [211G > A]**: Three individuals with *de novo* variants have been reported (Whitman et al., 2016). All had unilateral or asymmetric CFEOM, two had nystagmus, one had mild orbicularis oculi weakness, and one had sensorineural hearing loss. None had facial dysmorphisms, facial weakness, or microcephaly. All had intellectual disability and motor delay. While two had scoliosis, peripheral neuropathy was absent with the oldest individual reported at age 23 years. MRI revealed atypical cortical folding patterns with areas of increased and abnormal gyration and frontal lobe interdigitation in all three, and incomplete hippocampal rotation in two. There was a thin to absent corpus callosum, paucity of white matter, malformed basal ganglia, mild vermian hypoplasia, brainstem asymmetry, and CN III hypoplasia. Although none was diagnosed with Kallmann syndrome, a nine-year-old boy had unilateral olfactory sulci hypoplasia and he and his five-year-old sister both had an aberrant course of the olfactory nerves of unknown clinical significance. The individual with hearing loss also had hypoplasia or absence of the cochlear and inferior vestibular nerves bilaterally.

**TUBB3 G98S Syndrome [292G > A]**: Four unrelated individuals with *de novo* variants have been reported (Whitman et al., 2016; Smith et al., 2020; Stutterd et al., 2020; Stranneheim et al., 2021). One subject was described only as a rare disease patient without detailed phenotyping. Of the remaining three, one had unilateral CFEOM and nystagmus, one had strabismus with unilateral ptosis, and one had only exotropia. At least two had developmental delay, two had optic anomalies and one had microcephaly. MRI revealed a spectrum of MCDs including abnormal gyration, bilateral perisylvian polymicrogyria, and global polymicrogyria, as well as hypoplasia of the olfactory sulci, cerebellar vermis, and brainstem. CN III was bilaterally hypoplastic in the subject described with CFEOM. As with G71R, no one had been diagnosed with Kallmann syndrome, but two had abnormalities of the olfactory system on imaging.

### TUBB3 nonCFEOM/nonMCD variants

Several *TUBB3* variants have been reported to result in neurodevelopmental phenotypes without CFEOM or MCD (Figure 4 and Table 3). Most cause basal ganglia and cerebellar malformations while some are more difficult to categorize. While most are single reports, a few are recurrent.

**TUBB3 R46W [136C > T]**: Two boys, one documented *de novo*, have been reported (Froukh, 2017; Wiszniewski et al., 2018). An 11-year-old boy harboring a *de novo* variant was born at 6 months gestational age. He had ‘vertical’ strabismus with divergent squint (not defined as CFEOM) microcephaly, and a single seizure. The second child was normocephalic and had no history of seizures. Both had developmental and intellectual delay, ataxia, and spasticity. MRI of the second boy revealed corpus callosal agenesis, basal ganglia asymmetry, and reduced white matter volume without cerebellar malformations. He also had nodular heterotopy without notation of location or number; while this could indeed be a subcortical band heterotopia and part of the MCD spectrum, periventricular nodular heterotopia is far most common and is not considered an MCD. Ascertainment of additional cases should help determine if this variant should be reclassified as TUBB3-MCD and/or TUBB3-CFEOM.

**TUBB3 V175L [523G > C]**: A *de novo* variant in a severely impaired infant girl who died under comfort care at 1 month of age (Powis et al., 2018). A 19-week gestational age ultrasound revealed ventriculomegaly/hydrocephalus. Following birth, she had optic nerve pallor, central optic nerve excavation, abnormal grimacing, rhythmic extremity shaking, abnormal EEG with subclinical seizures, elevated CK, and no other spontaneous movements or eye opening. Brain CT showed severely dilated lateral ventricles, vermian hypoplasia and a Z-shaped brainstem. A Walker Warburg syndrome gene panel was uninformative and from trio exome only the *de novo TUBB3* variant was felt consistent with the infant’s presentation.

**TUBB3 T238A [712A > G]**: A *de novo* variant in a seven-year-old female with asymmetric pyramidal signs, delayed development and borderline intellectual disability (Romaniello et al., 2017). She was normocephalic with no neuro-ophthalmological symptoms or epilepsy. MRI revealed malformed corpus callosum and basal ganglia, unilateral malrotation of a cerebellar hemisphere, and a hypoplastic pons with a ventral cleft. The cerebral cortex appeared normal.

**TUBB3 V255I [763G > A]**: A 21-month-old boy and a 16-year-old girl, both *de novo*, have been reported (De Boer et al., 2022; Xue et al., 2022). Both have developmental delay with small head circumferences. The boy had elliptical pupils, photophobia, normal eye movements, seizures and, on MRI, a thin, hypoplastic corpus callosum. The teenager had convergent strabismus, minor facial dysmorphisms, mild pectus carinatum, mild nipple retraction, scoliosis, behavioral issues, sleep disturbances, and phonophobia. Her MRI revealed a hypoplastic corpus callosum, reduced white matter, and basal ganglia and cerebellar malformations. V255I has also been reported in TUBB4A (causing isolated hypomyelination).

**TUBB3 Q292E [874C > G]**: A *de novo* variant in an eight-month-old normocephalic boy with delayed development, axial hypotonia and distal hypertonia (Romaniello et al., 2017). Neuro-ophthalmological findings were not available. MRI revealed thin corpus callosum, basal ganglia malformations, hypoplastic cerebellar vermis, and hypoplastic and asymmetric pons.

**TUBB3 R391L [1172G > T]**: *De novo* in a 14-year-old girl and a 5-year-old boy (Costain et al., 2019; Ziats et al., 2020). Both had seizures and global developmental delay. The boy also had cortical visual impairment. MRI of the girl revealed thin corpus callosum and caudothalamic cysts, and of the boy revealed corpus callosal agenesis.

### *TUBB3* loss-of-function (LOF) and copy number variants (CNVs)

It remains unclear whether *TUBB3* loss-of-function (LOF) or copy number variants (CNVs) cause human disease.

***TUBB3* loss of function**: No human disorders resulting from *TUBB3* loss-of-function variants have been reported in fetuses or live-born individuals.

***TUBB3* deletions**: There are no reports that heterozygous or homozygous deletions of *TUBB3* result in a human phenotype. There is one report (Grønborg et al., 2015) of monozygotic twins harboring a *de novo* 0.32 Mb heterozygous deletion of 11 genes over 16q24.3, including *TUBB3* and 2 additional OMIM Morbid genes (*FANCA* and *MCIR*). The twins had developmental delay, mild lower limb spasticity, secondary microcephaly and slightly delayed myelination on MRI, but none of the callosal, basal ganglia, cerebellar, or brainstem anomalies or ocular phenotypes commonly seen with established *TUBB3* pathogenic variants.

In addition, ClinVar contains five heterozygous contiguous gene deletions containing *TUBB3* (National Center for Biotechnology Information. ClinVar; [VCV000058271.2]^3^ (accessed May 10, 2023) and, using the same format, VCV000058272.2, VCV000034317.2, VCV000615204.2, VCV000253762.1), all different from Grønborg et al. (2015), that are classified as of uncertain significance, benign, or likely benign. There are also three smaller heterozygous deletions within the *TUBB3* gene. Two are of unknown significance: a c.58-19del in intron 1 (VCV000510637.1) which is in gnomAD with allele frequency (AF) 0.00004 and as dbSnP rs755935820, and a heterozygous c.899del, p.M300Rfs*12 in exon 4 (VCV002230732.1). The third, classified as pathogenic by the clinical laboratory, is a heterozygous in-frame deletion of one amino acid in exon 4, c.572_574del, p.Q191del (VCV001695893.1) in a child with motor delay, hereditary episodic ataxia, vertigo, paroxysmal dystonia, epileptiform activity, sleep disturbance and anxiety. These data suggest that heterozygous loss of *TUBB3* function may be benign, similar to the lack of phenotype in *Tubb3* knock-out mice (see below). Interestingly, however, TUBB3 is strongly intolerant to heterozygous loss of function in the gnomAD database and thus it may have a role in humans distinct from that in mouse that results in early fetal loss.

***TUBB3* duplications**: There are 48 contiguous gene duplications involving *TUBB3* listed in ClinVar. They are all rather large (starting at 495 kb and encompassing at least 20 genes), and while 29 of them are classified as pathogenic and 5 as likely pathogenic, there is generally insufficient data to make any genotype/phenotype correlations. Thus, currently we lack strong evidence that duplication specifically of *TUBB3* results in human disease. ClinVar also lists one single base-pair duplication in intron 1, c.58-20dup, classified as benign, with an AF of 0.0232 and 146 homozygous individuals in the gnomAD database (VCV001175481.2). Similar to the intragenic deletions, this would be expected to result in a truncated protein.

This lack of evidence for pathogenic effects of *TUBB3* CNVs is supported by the *TUBB3* entry in the ClinGen showing no effects of haploinsufficiency or triplosensitivity (Clinical Genome Resource^4^ [6/30/2023]).

## Mechanisms underlying human TUBB3 developmental disorders

Investigators have examined how *TUBB3* variants alter tubulin heterodimer formation and incorporation into microtubules, motor and MAP interactions and trafficking, microtubule dynamics, and *in vivo* brain development in mouse and other model organisms. These mechanisms have been explored more extensively for *TUBB3* loss-of-function and TUBB3-CFEOM syndromes than for TUBB3-MCD syndromes, as detailed below.

### TUBB3 R262C knock-in mouse model

A mouse line harboring the Tubb3 R262C substitution (Tubb3^R262C^) is the only germline TUBB3 disease model reported to date (Tischfield et al., 2010). While this TUBB3-CFEOM model did not have a phenotype detected in the heterozygous state, homozygous mice died from respiratory compromise within hours of birth. TUBB3^R262C/R262C^ embryos had a CFEOM endophenotype that encompassed but was more extensive than the CFEOM endophenotype found in a *Kif21a* mouse model (Cheng et al., 2014); in the TUBB3^R262C/R262C^ embryos, the superior division of the oculomotor nerve failed to form, the trochlear nerve (CN IV) stalled, and some axons in the oculomotor nerve followed an aberrant course, sending aberrant axons toward the trochlear muscle target. The embryos also had brain pathology that recapitulated the more severe TUBB3-CFEOM phenotypes; the anterior commissure and corpus callosum were absent or thin compared to WT littermates. Importantly, TUBB3^R262C/+^ and TUBB3^R262C/R262C^ embryos did not show evidence of cortical cell migration defects and had normal neocortical layering.

Importantly, TUBB3 makes up only ~20–40% of neuronal β-tubulin (Latremoliere et al., 2018) and thus, in the heterozygous disease state, mutant TUBB3 isotypes would constitutes only 10–20% of neuronal β-tubulin. Thus, when proposing TUBB3-CFEOM and -MCD disease mechanisms, it is important to consider that, even if the mutant TUBB3 heterodimers were fully incorporated, they would constitute only one of every five to ten heterodimers.

### TUBB3 knock-out and knock-down mouse models

*Tubb3* knock-out mice (*Tubb3^−/−^*) were reported to be viable with no detectable neuroanatomical or behavioral defects and, in dorsal root ganglia (DRG) neurons, upregulation of most other β-tubulin isotypes resulted in wild-type pan-β-tubulin levels (Latremoliere et al., 2018). Notably, however, cultured DRG neurons had 22% slower outgrowth and, following sciatic nerve crush injury, axon regrowth of *Tubb3^−/−^* mice was 22% slower than wildtype littermates. *Tubb3* knockdown in mice *in vivo* and in cell culture yielded data generally consistent with *Tubb3^−/−^* data (Saillour et al., 2014). Acute knockdown of *Tubb3* in E14.5 mouse cortex resulted in a transient delay in radial migration of cortical neurons that fully recovered by E18.5, and the delay in migration was not fully rescued by overexpression of TUBB2B, TUBB1, or TUBB4A. These data suggested that *TUBB3* was not essential for central or peripheral nervous system development in mice.

### Altered tubulin heterodimer formation and incorporation into microtubules

The initial subset of reported TUBB3-CFEOM variants was examined for altered heterodimer formation and microtubule incorporation (Tischfield et al., 2010). Following expression in a rabbit reticulocyte lysate, transcription and translation were normal, but the yield of native heterodimers was very low for R62Q, R262C, A302T, and R380C and intermediate for R262H, E410K, D417H and D417N; notably, in general, the former substitutions result in milder and the latter in more severe phenotypes. When these mutants were expressed in Hela cells, R262C and R62Q, which result in isolated CFEOM, had generally lower levels of incorporation in microtubules compared to D417N, D417H, E410K, R262H, R380C, and A302T, which result in syndromic CFEOM and were similar to wild type. Together, the absence of a phenotype in *TUBB3* knockout mice, the isolated CFEOM phenotype found with variants that have poor heterodimer formation and lower microtubule incorporation, and the more severe phenotypes found with variants that form heterodimers and incorporate into microtubules support an altered- or gain-of-function disease mechanism that is dependent on mutant TUBB3 incorporation into microtubules.

The initial TUBB3-MCD variants were examined in a similar fashion. While transcription and translation of T178M, E205K, A302V, M323V and M388V were like wild type, the yield of native heterodimers was very low for A302V, M388V and E205K, intermediate for M323V, and near WT for T178M. Despite this, the mutant heterodimers incorporated into microtubules with only a subtle impairment of E205K and M388V (Poirier et al., 2010).

### Mapping human variants to the 3D TUBB3 structure

TUBB3-CFEOM variants are skewed toward altering residues in TUBB3 helix H12, where kinesins and dynein interact, and in a residue that interacts with H12, R262 (Figure 4). Remarkably, the amino acid substitutions E410K, D417N, D417H, and E421D alter the three negatively charged amino acid residues on the external surface helix H12, while R262C and R262H alter a residue positioned laterally to and below helix H12 and are predicted to break a hydrogen bond between R262 and D417.

By contrast to these six variants, R380C alters an amino acid residue on the external surface of helix H11 where MAPs are proposed to interact, while A302T, in the S8 loop following H9, alters a residue positioned laterally and below helix H11 (Tischfield et al., 2010, 2011).

Beyond the TUBB3-CFEOM variants in H11 and H12 are (1) R62, which is located in a loop mediating lateral interactions but has only been reported one in one individual with isolated CFEOM and his unaffected mother, and (2) S78 which clusters with G71 and G98 near the E site of the GTP binding and has also been reported only once in a parent and child with CFEOM. Thus, these two variants may not be pathogenic.

By contrast, TUBB3-MCD and TUBB3-nonCFEOM/nonMCD variants exclude helices H11 and H12 with the exception of M388, which falls at the end of H11 and has been reported with DN changes in a fetus and a deceased infant. Otherwise, the TUBB3-MCD and TUBB3-nonCFEOM/nonMCD variants exclude the C-terminus of the protein and instead are distributed throughout the N-terminal and intermediate domains that regulate GTP binding, heterodimer stability, and longitudinal and lateral interactions (Figure 4; Löwe et al., 2001; Nogales and Wang, 2006).

Finally, G71R and G98S, which cause both CFEOM and MCD (Whitman et al., 2016) are located in the N-terminal domain H2 and T3, near the E site of the GTP binding pocket, and close to S78T (Figure 4). These three variants are spatially distinct from previously reported substitutions and their mechanisms have not been defined.

### Kinesins interact with negatively charged residues in microtubule helix H12

Kinesin and dynein motor proteins use microtubules as tracks for motility, and kinesins interact with the alpha helix H12 of both α- and β-tubulin (Hunter and Allingham, 2020). Data support the interaction of kinesins with three of the four negatively charged amino acid residues in helix H12 of β-tubulin. In a yeast expression system, E410A, D417A, and E421A (but not E412A) led to a decrease in the microtubule affinity for kinesin (Uchimura et al., 2006). In *C. elegans*, KLP-12, the KIF21A/B homolog, interacted with E410 of β-tubulin H12 as well as with several sites on α-tubulin (Nogales and Wang, 2006; Taguchi et al., 2022).

### TUBB3 variants altering residues in or interacting with H12, but not H11 or elsewhere, reduce kinesin-microtubule interactions

Remarkably, the TUBB3-CFEOM variants located on H12 alter the negatively charged residues E410, D417, and E421, and R262 which is predicted to interact with D417. This observation led to the examination of microtubule-kinesin interactions in yeast. H12 TUBB3-CFEOM amino acid substitutions E410K, D417N, and D417H, and H12-interacting substitution R262C were introduced into Tub2p, the single β-tubulin isotype in budding yeast, and the interaction of WT and mutant microtubules with two processive yeast plus end directed kinesins, Kip3p and Kip2p-microtubule, examined. Each resulted in a significant decrease in kinesin accumulation at the cytoplasmic microtubule plus ends compared to wildtype (Tischfield et al., 2010). Subsequent generation and polymerization of purified human tubulin heterodimers containing WT, E410K, or D417H TUBB3 were similarly found to reduce binding of kinesin-1 and kinesin-5B motor proteins as well as PRC1 and NuMA to mutant TUBB3 microtubules compared to WT (Ti et al., 2016), and R262H TUBB3 was found to reduce KIF5B binding, processivity, and ATPase activity (Minoura et al., 2016). Finally, pelleting microtubules from WT and TUBB3^R262C/R262C^ mice revealed that TUBB3^R262C/R262C^ microtubules had reduced KIF21A binding affinity compared to WT (Tischfield et al., 2010).

By contrast, the TUBB3-CFEOM substitution on H11 (R380C) and elsewhere within the microtubule (R62Q, A302T) resulted in no change (R62Q, A302T) or increased Kip3p accumulation (R380C) on yeast astral microtubules (Tischfield et al., 2010).

Consistent with these data, mutant and WT TUBB3 constructs have also been overexpressed in cultured hippocampal neurons to test the effects of TUBB3-CFEOM substitutions (R262C, E410K, D417H, R380C, R62Q) and MCD substitutions (G82R, T178M, A302V, M323V, M388V) on axonal transport of Rab3a (Niwa et al., 2013). Interestingly, only microtubules with H12 TUBB3-CFEOM substitutions E410K and D417H had reduced axonal transport, while incorporation of the remaining CFEOM and MCD mutant isotypes resulted in transport similar to WT. The authors also found that the E410K and D417H microtubules had reduced accumulation of isolated kinesin motors KIF1A, KIF1B, KIF5 and KIF21A at the distal processes of transfected hippocampal neurons and reduced mitochondrial transport in transfected DRG neurons compared to wildtype neurons (Niwa et al., 2013).

### TUBB3 increases microtubule catastrophe and dynamic instability *in vitro* and *in vivo*, and TUBB3 variants alter this function

The incorporation of TUBB3 into microtubules has been long known to increase dynamic instability (Panda et al., 1994). *In vitro*, microtubules composed of purified TUBA1B/TUBB3 isotypes depolymerize faster than those composed of TUBA1B/TUBB2B isotypes (Ti et al., 2018), while having similar growth rates (Pamula et al., 2016). Moreover, when the C-terminal tail of TUBB1 is substituted for the C-terminal tail of TUBB3 in purified tubulin preparations or in cultured neurons, the resultant microtubules are more stable than those expressing WT TUBB3 protein (Pamula et al., 2016; Parker et al., 2018). These data, in combination with other reports (Vemu et al., 2016, 2017) support the general hypothesis that the TUBB3 isotype increases microtubule catastrophe frequency and dynamic instability. These data also suggest that the ends of microtubules containing TUBB3 heterodimers are less stable than those assembled from alternative beta-tubulin isotypes, and TUBB3 can exert this effect on microtubule end stability even when present as a minority component of a blend of different heterodimers. This is notable, given the relatively low expression of TUBB3 in cells and that TUBB3 genetic disorders are dominant, altering only 50% of TUBB3 in the neuron.

DRG neurons cultured from *Tubb3^−/−^* mice had enlarged growth cones with decreased microtubule dynamics and both central and peripheral neurons had post-translational markers associated with increased stability (Latremoliere et al., 2018). *Tubb3* knockdown was also demonstrated to stabilize microtubules in cultured hippocampal neurons and increase KIF5C-mediated transport (Radwitz et al., 2022). These data indicated that, at least in some neuronal cell types, TUBB3 increases microtubule dynamic instability and neurite outgrowth *in vivo* (Latremoliere et al., 2018).

Multiple studies support the stabilization of neuronal microtubules by TUBB3-CFEOM variants, similar to loss of the TUBB3 isotype. Insertion of the TUBB3-CFEOM human variants into yeast β-tubulin results in variable degrees of benomyl resistance and stable to highly stable microtubules (Tischfield et al., 2010). Live imaging of yeast astral microtubules in G1 phase revealed that R262C, R262H, and E410K astral microtubules were often long and grew at a decreased rate of polymerization followed by a more rapid and complete catastrophe without recovery. By contrast, A302T, R62Q, and R380C substitutions resulted in more stable microtubules with significantly diminished overall dynamics; microtubules had longer lifetimes, spending most of the time in prolonged paused states, with reduced rates of polymerization and depolymerization (Tischfield et al., 2010). In a different set of experiments, purified human microtubules harboring TUBB3-CFEOM R262H and D417H variants were examined (Ti et al., 2016). Similar to the yeast data, both mutant microtubules underwent catastrophe less frequently than WT at both the plus and minus ends and were less sensitive to the microtubule depolymerizing drug colchicine. In contrast to the yeast data, microtubules harboring the mutant TUBB3 D417H isotype had an increased polymerization rate at both the plus and minus ends, while the polymerization rate of microtubules harboring the mutant R262H isotype was unchanged. Despite some differences, both studies support TUBB3-CFEOM variants as reducing microtubule dynamic instability, similar to the effect following loss of TUBB3 *in vitro* and *in vivo*. In addition, the reduced affinity of D417H tubulin heterodimers for GST-tagged tumor overexpression gene (TOG) domains and colchicine suggested that introduction of the TUBB3-CFEOM variant may reduce the fraction of curved versus straight tubulin heterodimers compared to wildtype (Ti et al., 2016).

The effect of TUBB3-MCD variants on microtubule dynamics has been studied in T178M and E205K patient fibroblasts, which were reported to have decreased microtubule resistance to cold treatment compared to controls, suggesting that the TUBB3-MCD mutant microtubules were less stable than WT (Poirier et al., 2010). While the stabilizing effects of the TUBB3-CFEOM variants versus the destabilizing effects of the TUBB3-MCD variants were initially hypothesized to account for the differences in TUBB3-CFEOM versus TUBB3-MCD phenotypes, newer data do not support this. First, a more recent study introduced the T178M variant into yeast and found that microtubules harboring the heterozygous tub2-T178M substitution spent more time paused, with ‘attenuated’ microtubules that were neither growing nor shortening, compared to wildtype, while the homozygous substitution was lethal to yeast (Park et al., 2021). These results in yeast suggest that the T178M substitution does not increase microtubule dynamics, but instead blocks tubulin’s ability to switch between activated and deactivated states, which is an underlying feature of dynamic instability. Second, there are now reports of TUBB3 variants that cause both CFEOM and MCD (Whitman et al., 2016); it will be interesting to determine the effect on dynamic instability of these rare variants.

### TUBB3 post-translational modifications (PTMs) alter wildtype microtubule function

Cells generate microtubule diversity, in part, through reversible PTMs as well as by the expression of different tubulin isotypes (Janke, 2014; Janke and Magiera, 2020). β-tubulins undergo PTM of their C-terminal tails, the sequence with greatest divergence between isotypes. There is *in vitro* evidence that PTMs of TUBB3 can provide isotype-specific regulation of MAP and motor behaviors, and this may occur, in part, because of TUBB3’s unique terminal lysine; the C-terminal tails only of TUBB3, and of TUBB1 which is expressed in hematopoietic cells, contain a positively charged lysine. Polyglutamylation adds negative charge to the C-terminal tail and, in brain β-tubulin, increased microtubule binding affinity for MAPs and impacted kinesin function (Szczesna et al., 2022; Genova et al., 2023). When human TUBB3 or TUBB2B C-terminus was fused with yeast β-tubulin, the run length of KIF5 was less on TUBB3 compared to the TUBB2B hybrid microtubules, while KIF17 and dynein run lengths did not differ. Removing either the terminal lysine or polyglutamylation of the TUBB3 C-terminal tail restored KIF5B run length to that of TUBB2B (Sirajuddin et al., 2014). Similarly, KIF1A interacted with TUBB3 C-terminal tail, and polyglutamylation of TUBB3 reduced KIF1A pausing and increased its landing rate and run length (Lessard et al., 2019).

There is limited *in vivo* data regarding the relationship of TUBB3 C-terminal tail and PTMs. *Tubb3* knockout mice had increased polyglutamylation of brain and sciatic nerve microtubules (Latremoliere et al., 2018). By contrast, following TUBB3 knockdown in cultured hippocampal neurons there was decreased polyglutamylation in the axon and increased velocity of KIF5C (Radwitz et al., 2022). Finally, one early report suggested that TUBB3 phosphorylation was correlated with neurite outgrowth in PC12 cells (Aletta, 1996). There is no published data addressing whether TUBB3-CFEOM or -MCD variants alter PTMs.

### TUBB3 can influence wildtype microtubule protofilament number

Protofilament number regulates the width of the microtubule and the configuration of the tracks for intracellular cargo transport (Chaaban and Brouhard, 2017) and may contribute to dynamic instability (Lee et al., 2021). Protofilament number is a function of the inter-protofilament angle which, itself, is determined by tubulin isotypes, nucleation factors, and PMTs (Chaaban and Brouhard, 2017). In *C. elegans* and *Drosophilia*, loss or ectopic expression of specific β-tubulin isotypes changed protofilament number and, in some cases, altered microtubule dynamics (Chalfie and Thomson, 1982; Raff et al., 1997; Zheng et al., 2017; Lee et al., 2021).

In eukaryotes, the canonical microtubule is composed of 13 protofilaments (Tilney et al., 1973; Unger et al., 1990). *In vitro*, purified TUBA1B/TUBB3 heterodimers formed canonical 13 protofilament microtubules; by contrast, purified TUBA1B/TUBB2B heterodimers formed 14 and/or 15 protofilament microtubules (Ti et al., 2018). These data support the role of TUBB3 in the formation of 13-protofilament microtubules, but it is not known whether loss of TUBB3 or the presence of TUBB3-CFEOM or -MCD variants alter protofilament number.

## Human *KIF21A* variants

Below, we review the phenotypes reported with specific *KIF21A* variants, as summarized in Figure 5 and Table 4.

**Table 4:** KIF21A-CFEOM variant phenotypes.

NM_006086	Variant	C28W	D352E	F355S	M356T	L685P	E944Q	M947V	M947T	M947R	M947I	R954W	R954Q	R954L	D1001del	A1008P	I1010T
	Nucleotide	84 C > G	1056 C > G	1064 T > C	1067 T > C	2054 T > C	2,830 G > C	2839 A > G	2840 T > C	2840 T > G	2841 G > A	2860 C > T	2861 G > A	2861 G > T	3000_3002delTGA	3022 G > C	3029 T > C
Pedigree Information	# Pedigrees^1^	1 (Lu)	1	1	4	1	2	1	2	1	1	50+	17	3	1	1	2
	# M/F/fetus	5/2/0	0/1/0	1/0/0	10/11/1	1/0/0	3/0/0	0/3/0	3/0/0	0/1/0	15/12/0	25+/25+/0	11/24/0	2/3/0	1/2/0	0/1/0	14/8/0
	Inheritance	AD	DN	DN	AD+DN	DN	AD+DN	AD	AD+DN	DN	AD	AD+DN	AD+DN	AD+DN	AD	DN	AD+DN
	Eldest	73y	10y	child	70s	13y	adult	adult	adult	36y	adult	adult	adult	adult	adult	child	adult
Peripheral NS dysfunction	Kallmann	nr	nr	nr	no	nr	no	no	no	no	no	no	no	no	nr	no	no
	CN II	nr	nr	nr	no	nr	no	no	no	no	no	no	no	no	51–75%	no	no
	CN III: CFEOM	100%	yes	yes	100%	yes	100%	100%	100%	100%	75–100%	75–100%	100%	100%	100%	100%	100%
	CN VII	nr	yes	yes	no	yes	no	no	mild	no	no	no	no	no	nr	no	mild
	CN IX, X	nr	nr	nr	no	nr	no	no	no	no	no	no	no	no	nr	no	no
	CN X	nr	nr	nr	no	nr	no	no	no	no	no	no	no	no	nr	no	no
	PN	nr	nr	nr	no	yes	no	no	no	no	no	no	no	no	nr	no	no
	C contractures	nr	nr	nr	no	nr	no	no	no	no	no	no	no	no	nr	no	no
	Cyclic vomiting	nr	nr	nr	no	nr	no	no	no	no	no	no	no	no	nr	no	no
	Cardiac aryth	nr	nr	nr	no	nr	no	no	no	no	no	no	no	no	nr	no	no
Central NS dysfunction	Micro-cephaly	nr	nr	nr	no	no	no	no	no	no	no	no	no	no	nr	no	no
	Strabismus	nr	nr	nr	no	nr	no	no	no	no	no	no	no	no	nr	no	no
	Nystagmus	nr	nr	nr	no	nr	no	no	no	no	no	no	no	no	nr	no	no
	OMA	nr	nr	nr	no	nr	no	no	no	no	no	no	no	no	nr	no	no
	Seizures	nr	nr	nr	no	nr	no	no	no	no	no	no	no	no	nr	no	no
	Motor delay	nr	yes	yes	no	nr	no	no	no	no	no	no	no	no	nr	no	no
	LD	nr	L NA	L NA	no	L NA	no	no	no	no	no	no	no	no	nr	no	no
	ID/Social	nr	yes	yes	no	yes	no	no	no	no	no	no	no	no	nr	no	no
Brain MRI abnormality	Cerebral cortex	nr	no	nr	no	no	no	no	no	no	no	no	no	no	nr	no	no
	AC	nr	no	nr	no	nr	no	no	no	no	no	no	no	no	nr	no	no
	CC	nr	no	yes	no	yes	no	no	no	no	no	no	no	no	nr	no	no
	White matter	nr	no	nr	no	yes	no	no	no	no	no	no	no	no	nr	no	no
	Basal ganglia	nr	no	nr	no	yes	no	no	no	no	no	no	no	no	nr	no	no
	Cerebellum	nr	no	nr	no	yes	no	no	no	no	no	no	no	no	nr	no	no
	Brainstem	nr	no	nr	no	yes	no	no	no	no	no	no	no	no	nr	no	no

^1^Corresponding references for key pedigrees provided in the main text. no, absent; nr, not reported or mentioned in publication(s); percentages, percentage range of affected individuals for whom the phenotype has been reported. # Pedigrees, number of pedigrees reported in the literature; #M/F/U/fetus, number of affected individuals who are male, female, unknown or were a fetal case. AD, autosomal dominant; DN, *de novo*. Eldest, eldest reported individual to date harboring the variant. Y, years of age. Kallmann, anosmia and hypogonadotropic hypogonadism and/or hypoplasia/absence of olfactory sulci and bulbs. CN II: ONH, optic nerve: optic nerve hypoplasia. CN III: CFEOM, oculomotor nerve: congenital fibrosis of the extraocular muscles. CN VII: FW, facial nerve: congenital facial weakness. CN IX-XII: Sw., lower cranial nerves: swallowing dysfunction. CN X: VC, vagal nerve: vocal cord paralysis. PN, mixed sensorimotor axonal polyneuropathy. C contractures, congenital contractures. Cardiac Arrhy, sick sinus syndrome with or without a pacemaker. S NA, strabismus not relevant given CFEOM. N NA, nystagmus not relevant given CFEOM. OMA, oculomotor apraxia. LD, learning disorder. LD NA, LD not relevant given ID/Social disabilities. ID/Social, intellectual and social disabilities (including ASD). Cerebral cortex, malformation of cortical development. AC, hypoplasia or absence of anterior commissure. CC, hypoplasia or absence of corpus callosum. White matter, paucity of central white matter and/or aberrant fascicles or signal where noted. Basal ganglia, presence of any abnormality ranging from mild asymmetry of caudate to full malformation. Cerebellum, malformation of vermis and/or hemispheres. Brainstem, hypoplasia or malformation.

### Isolated KIF21A-CFEOM

KIF21A C28W [84C > G], M356T [1067 T > C], E944Q [2830G > C], M947V [2839A > G], M947T [2840 T > C], M947R [2840 T > G], M947I [2841G > A], R954W [2860C > T], R954Q [2861G > A], R954L [2861G > T], D1001del [3000_3002delTGA], A1008P [3022G > C], I1010T [3029 T > C] (Yamada et al., 2003, 2004, 2005; Chan et al., 2007; Lu et al., 2008; Yang et al., 2010; Wang et al., 2011; Al-Haddad et al., 2021; Moon et al., 2021; Jia et al., 2022; Chen et al., 2023). These heterozygous missense variants in *KIF21A* occur in association with CFEOM and were first reported in 2003 (Yamada et al., 2003). Many AD pedigrees and *de novo* cases harboring these recurrent variants have been reported subsequently. Remarkably, these variants alter only eight amino acid residues located in two distinct regions of the KIF21A protein: two are in the motor domain and six are within a 66 amino acid span of the third coiled-coil stalk domain. Among these, R954W accounts for at least 75% of KIF21A-CFEOM cases (Table 4 and Figure 5).

Individuals harboring KIF21A-CFEOM variants most typically have isolated bilateral CFEOM (Engle et al., 1997) with ptosis, hypotropic eyes, inability to elevate either eyes above midline, and variably restricted horizontal movements. Affected individuals often have aberrant residual eye movements, most commonly synergistic convergence on attempted upgaze, and a compensatory chin-up head position. While bilateral, there is variability in the degree of infraduction, the horizontal position of the eyes at rest, and the degree of normal and aberrant movements. MRI revealed hypoplasia of the oculomotor nerve and, in some cases, the abducens nerve, and hypoplasia of EOM. The levator palpebrae superioris and superior rectus complex are always, the medial and inferior recti are often, and the lateral rectus is occasionally hypoplastic (Demer et al., 2005).

These twelve missense variants and one in-frame amino acid deletion almost always result in fully penetrant, indistinguishable clinical phenotypes. While some affected individuals are reported to have mild facial weakness, it remains unclear if this is true and may be confused with the appearance of the face in the chin-up head position. Infants and young children may also have mild hypotonia and gross motor delays, but these normalize over time. In addition to these caveats, three exceptions are reported. M947I, reported in one large consanguineous Turkish pedigree, included eighteen affected members with classic CFEOM, eleven with eye(s) in the neutral primary position, residual upgaze, and/or absence of ptosis, and one non-penetrant carrier (Sener et al., 2000). In addition, affected members of a consanguineous Saudi pedigree who segregate the common R954W substitution had some residual upgaze (Yamada et al., 2004), and several members of a dominant family that also segregate R954W have CFEOM, a later-onset gait deterioration, and cerebellar atrophy (Fabio et al., 2013).

### Syndromic KIF21A-CFEOM

While the variants above result in isolated CFEOM, there are three reports of novel *KIF21A* variants in pedigrees that have additional features even more reminiscent of the TUBB3-CFEOM syndromes (Table 4 and Figure 5).

**KIF21A D352E (1056C > G)**: A *de novo* variant in a 10-year-old Hispanic girl with classic CFEOM, muscle weakness, and motor and cognitive developmental delay. Brain MRI and DTI were reportedly normal with thin EOM (Ali et al., 2013).

**KIF21A F355S (1064 T > C)**: A *de novo* variant in a Chinese boy with CFEOM, facial weakness, frontal bony prominence, and delayed developmental milestones. MRI revealed hypoplasia of the corpus callosum, levator/superior rectus EOM complex, and facial nerves (Jia et al., 2022).

**KIF21A L685P (2054 T > C) (also known as pL672P due to a difference in cDNA numbering)**: A *de novo* variant in a 13-year-old boy with exotropic CFEOM, asymetrical congenital facial weakness, and axonal sensory-motor peripheral neuropathy with onset prior to 11 years of age (Soliani et al., 2020). At thirteen years-of-age, he walked with lower-limb ankle-foot orthoses. He had intellectual disability with normal head circumference, social, and communication skills. MRI revealed thin corpus callosum, small caudate bodies, reduced cerebral white matter, cerebellar vermian and hemisphere hypoplasia, dysmorphic midbrain, and small superior, inferior, and medial recti EOM. This variant is in a unique location and is remarkable in closely phenocopying TUBB3-CFEOM variants.

### *KIF21A* loss-of-function (LOF) and copy number variants (CNVs)

#### *KIF21A* homozygous loss-of-function variants cause fetal akinesia syndrome

Five fetuses with arthrogryposis of multiple joints, pulmonary hypoplasia, and facial dysmorphisms from two unrelated Turkish families harbored homozygous loss-of-function variants in *KIF21A* (Falb et al., 2021). The three fetuses from the first pedigree harbored a stop variant c.1346 T > A, (p.L449*) in exon 9, while the two fetuses from the second pedigree harbored a frameshift variant c.2371del, (p.R791Efs*8) in exon 17. Both are predicted to result in nonsense mediated decay of the truncated mRNA. Thus, unlike *TUBB3*, *KIF21A* appears to have a critical role in development. By contrast, their heterozygous parents were unaffected and, thus, *KIF21A* haploinsufficiency does not cause a recognized human phenotype.

#### KIF21A deletions

Three contiguous gene deletions containing *KIF21A* have been entered into ClinVar as VUS (variant of uncertain significance): chr12:39560669-43285298 (3.7 Mb), chr12:39792367-39965548 (173 kb) and chr12:38207168-40145174 (1.9 Mb) (VCV001809396.1, VCV001808114.1, VCV000563977.1). A fourth deletion, 1.0 Mb at 12: 39577315–40582797 is listed as pathogenic, but no supporting evidence is provided (VCV000687619.2); indeed, no conditions or phenotypes are listed to clarify the significance of these multi-gene deletions. Notably, none are present in the homozygous state, and the fetal akinesia syndrome, with lack of symptoms in the heterozygous carrier parents and healthy siblings, informs us that heterozygous loss of *KIF21A* does not manifest clinically.

ClinVar does not reference the non-frameshift deletion D1001del or LOF variants referenced above, but contains three intragenic deletions within *KIF21A*, only one of which is coding: (1) a 5 bp deletion in exon 36 (c.4602_4606del; p.T1535Qfs*3) classified by the reporting laboratory as a VUS (VCV000308534.5, VCV001710001.1, VCV001710001.1) reportedly in one female with CFEOM; (2) a one bp 3’ UTR deletion (c.*690del) reported out by the clinical lab as identified in an individual of unknown affected status for CFEOM, classified as benign with a gnomAD AF of 0.1377 and found homozygous in 156 cases in gnomAD; and (3) a single bp intron 3 deletion (c.451-6del), reported out by the clinical lab as identified in an individual of unknown affected status with an unspecified diagnosis, also benign with gnomAD AF 0.0007505 and twice in the homozygous state.

#### *KIF21A* duplications

ClinVar lists sixteen heterozygous CNVs due to contiguous gene duplications encompassing *KIF21A*, of which several particularly large duplications are classified as pathogenic or likely pathogenic, but do not provide sufficient detail to assess any association with KIF21A. ClinVar contains 4 heterozygous intragenic variants all classified as VUS (VCV000632188.4, VCV000308533.5], [VCV000308530.5, VCV000308559.5): (1) one frame-shift coding variant (c.3887dup; p.N1296fs) in an individual with unknown affection status, absent from gnomAD; (2) two single bp 3’ UTR duplications (c.*690dup and c.*845dup); and (3) a one bp duplication in intron 24 (c.3341–4_3,341–3dup). The ClinGen resource does not have any evidence regarding CNVs or dosage sensitivity classifications for *KIF21A* (Clinical Genome Resource^5^ [6/30/2023]).

## Mechanisms underlying human KIF21A developmental disorders

### KIF21A R954W knock-in mouse model

KIF21A^R954W^ knockin mice have a remarkably localized phenotype and recapitulate human CFEOM, with 42 and 92% penetrance of CFEOM in heterozygous and homozygous mice, respectively (Cheng et al., 2014). During embryonic development, oculomotor axons exited the brainstem but those destined to become part of the superior division stalled and formed a premature decision region followed by secondary axon retraction and motor neuron death. In explant cultures, KIF21A^R954W^ oculomotor axons had normal outgrowth but enlarged growth cones with increased number of filopodia.

### KIF21A knock-out mouse model

*Kif21a* knock-out mice die within hours of birth from respiratory compromise (Cheng et al., 2014). While human KIF21A-fetal akinesia variants are predicted to result in complete loss of KIF21A protein, how absence of KIF21A results in early postnatal death and how it may alter peripheral and central nervous system development in mice and humans remains to be elucidated.

### KIF21A-CFEOM variants attenuate KIF21A autoinhibition

KIF21A-CFEOM variants cause altered or gain of KIF21A function. KIF21A activity is controlled, at least in part, though autoinhibition; the lateral aspect of the motor domain interacts with the third coiled-coil domain of the stalk, and in this ‘closed’ conformation the interaction of KIF21A with microtubules is reduced or abolished (van der Vaart et al., 2013; Cheng et al., 2014). X-ray crystallography confirmed that the third coiled-coil domain interacts with the motor domain at a location close to C28 and M356 and at a distance from the ATP- and microtubule-interaction sites (Bianchi et al., 2016).

Remarkably, *KIF21A* missense variants that result in isolated CFEOM alter amino acid residues located precisely in the lateral aspect of the motor domain or the third coiled-coil domain of the stalk (Figure 5) and disrupt the interaction of these two domains, thus attenuating KIF21A autoinhibition (van der Vaart et al., 2013; Cheng et al., 2014). Single molecule studies of mutant KIF21A harboring a motor or third coiled coil variant resulted in an increased number of active landing events on microtubules compared to wildtype KIF21A, but no change in velocity or run length (Cheng et al., 2014). Similarly, in HeLa cells, KIF21A-R954W-GFP had processive motility along microtubule tracks while KIF21A-WT-GFP did not. In cultured hippocampal neurons, KIF21A-R954W-GFP and KIF21A-WT-GFP fluorescence recovery after photobleaching were similar, but there was greater growth cone accumulation of KIF21A-R954W-GFP (van der Vaart et al., 2013). These data are consistent with KIF21A-CFEOM amino acid substitutions resulting in attenuated KIF21A autoinhibition compared to wildtype.

The mechanism underlying the KIF21A L685P substitution, located in the second coiled-coil domain of the stalk, has not been reported. This variant, which results in more of a TUBB3-CFEOM phenotype with CFEOM, facial weakness, progressive peripheral neuropathy, and intellectual disability, may simply attenuate autoinhibition to a greater degree than the substitutions that result in isolated CFEOM, or may alter KIF21A function in another way. Given the similarity in phenotype to TUBB3-CFEOM, uncovering how this variant perturbs KIF21A function has the potential to provide a mechanistic link between KIF21A- and TUBB3-CFEOM.

### KIF21A stabilizes and stalls microtubules at the cell periphery, and this is enhanced by KIF21A-R954W overexpression

KIF21A has been shown to stabilize microtubules and to stall microtubule growth at the cell cortex and neuronal growth cones. The KIF21A motor domain alone can reduce microtubule growth rate and suppress catastrophes, resulting in stalled microtubules (van der Vaart et al., 2013). Similarly, the motor domain of KLP-12, the *C. elegans* homolog of KIF21A/B, represses microtubule polymerization *in vitro*.

KIF21A accumulates at the cell cortex of non-neuronal cells and the growth cone/synapse of neurons (van der Vaart et al., 2013; Cheng et al., 2014). At the cell cortex, the coiled-coil stalk domain distal to the coiled-coil containing the KIF21A-CFEOM1 variants interacts with the scaffold protein KANK1 (Kakinuma and Kiyama, 2009; van der Vaart et al., 2013; Pan et al., 2018). KANK1, in turn, interacts with both liprin-β1, a member of the cortical microtubule stabilization complex, and with talin, a component of the focal adhesion complex (Bouchet et al., 2016). Focal adhesions link the cell cortex with the extracellular matrix, provide structural support, and serve as signaling hubs critical to cell migration and axon growth and guidance. Thus, at least *in vitro*, KIF21A stabilizes microtubules and links the stabilized microtubules to the cortical microtubule stabilization complex and focal adhesions at the cell cortex.

Following overexpression of mutant KIF21A-R954W in cultured hippocampal neurons, axon length was found to be decreased and axonal branching increased, with smaller and less dynamic growth cones compared to wildtype, consistent with a gain-of-function phenotype (van der Vaart et al., 2013).

It remains to be determined how attenuation of KIF21A autoinhibition in all cells in which it is expressed leads to the isolated failure of ocular motor neuron development, and whether these phenotypes result from altered cargo transport, reduced microtubule dynamics, or both. Notably, however, microtubules play a critical role in cell migration and axon growth and guidance. Thus, perturbing the role that KIF21A plays in pausing microtubule growth at the cell cortex (and presumably within growth cones of developing neurons) as a result of gain- or loss of KIF21A function is an enticing explanation.

### Kinesin-4 family members contribute to tubulin heterodimer angle

The crystal structure of kinesin-4 family member KLP-12 complexed with tubulin revealed that KLP-12 introduces an intermediate curvature to the heterodimer angle at growing microtubule tips, inhibiting microtubule dynamics. This intermediate curvature falls between the straight conformation of the KIF5B-tubulin complex that results in microtubule polymerization, and the more curved conformation of the KIF2C-tubulin complex that results in microtubule catastrophe (Taguchi et al., 2022). This role of kinesin-4 family members in determining the heterodimer angle is an interesting parallel to the observation that TUBB3 D417H straightens the tubulin angle (Ti et al., 2016), and could provide a shared mechanism between TUBB3- and KIF21A-CFEOM. Thus, it will be interesting to determine if KIF21A contributes to heterodimer angle and if this angle is altered by KIF21A- and TUBB3-CFEOM variants.

### Insights from KIF21B function

KIF21B may provide insights into KIF21A function. KIF21B appears to be important for dendritic development in the central nervous system. *KIF21B^−/−^* mice had impaired learning and memory, decreased dendritic branching and spine number, and microtubules that grew more slowly and persistently (Muhia et al., 2016). Interestingly, KIF21B was shown to have dual motor and non-motor functions: it delivered BDNF–TrkB cargo retrogradely in dendrites while also regulating microtubule dynamics through its stalk domain (Ghiretti et al., 2016). In a cell-free system, KIF21B accumulated at microtubule plus-ends and induced pausing, and this function depended on separate microtubule binding domains in the KIF21B stalk and WD40 domains (Ghiretti et al., 2016; Riel et al., 2017). Neuronal activity enhanced KIF21B motility and reduced its role in microtubule dynamics (Ghiretti et al., 2016). In a separate study, the KIF21B coiled-coil stalk region homologous to the KIF21A autoinhibition domain did not fully inhibit KIF21B interaction with the microtubule, but instead enhanced pausing by preventing detachment from microtubule tips (Riel et al., 2017). It remains to be determined if KIF21A has a non-motor microtubule binding domain, if neuronal activity switches KIF21A activity from transport to regulation of microtubule dynamics, and whether KIF21A’s role in growth cones is similar to its role in focal adhesions of non-neuronal cells.

## Future directions

While remarkable progress has been made in our definition and understanding of the TUBB3- and KIF21A- syndromes, much about the role of these proteins in development and disease remains undefined.

It is not yet known why one set of TUBB3 variants primarily causes CFEOM syndromes, which are disorders of peripheral and, to a lesser extent, central axon growth and guidance, while a second set causes MCD syndromes, which are disorders primarily of neuronal identity, migration, and organization. Data suggest that both sets of variants stabilize microtubules, although additional studies are necessary to compare the effects of different variants on dynamic instability. It is also notable that the subset of TUBB3-CFEOM variants that map to helix H12 (or interact with it) reduces the interactions of kinesin motor proteins with microtubules, while none of the TUBB3-MCD variants have been shown to do so. Another subset of TUBB3-CFEOM variants, however, does not reduce kinesin interactions. This suggests that the alterations in kinesin-microtubule interactions by some variants may be upstream of a common mechanism or may be a secondary effect.

It is also not yet known if TUBB3-CFEOM and KIF21A-CFEOM variants share a common mechanism. Both alter microtubule dynamics and both may alter tubulin heterodimer angle. Notably, however, TUBB3-CFEOM variants located on helix H12 reduce, while KIF21A-CFEOM variants increase KIF21A-microtubule interactions. The recently reported KIF21A L685P substitution results in a syndromic CFEOM human phenotype that is very similar to the syndromic TUBB3-CFEOM phenotypes, with facial weakness, progressive axonal polyneuropathy, and intellectual disability in addition to CFEOM. Studies of how this variant alters KIF21A function may provide insight into shared TUBB3 and KIF21A disease mechanisms.

Finally, the basis of the selective vulnerability of the oculomotor nerve to *KIF21A* and *TUBB3*-CFEOM variants, despite the wide expression patterns of both genes, remains a mystery.

In addition to ongoing *in vitro* experiments, studies of existing and new *Tubb3* and *Kif21a* missense and knock-out mouse models should help to address many of these questions. Comparing the developmental pathologies of each *Tubb3* and *Kif21a* knock-in and knock-out mouse model to one another in isolation and in combination may shed light on whether they act through the same or parallel pathways both in the wildtype and mutant conditions. This could include comparative histology of brain and CN development of cleared embryos, and comparative growth properties of affected and unaffected neurons in dissociated culture, explant culture, and live imaging in oculomotor slice culture (Whitman et al., 2019). Cryo-EM could be pursued to determine wildtype and mutant protofilament number and heterodimer angles. For disease variants without mouse models, one could take advantage of techniques to differentiate wildtype and mutant embryonic stem cells (ESC) and induce pluripotent stem cells (iPSCs) to specific cranial motor neurons (Allodi et al., 2019; An et al., 2019). These approaches would also provide large numbers of cells for *in vitro* studies, and may eventually lead to successful brainstem motor neuron organoids for modeling. Together, these approaches will help address overarching questions not only about these rare Mendelian disorders, but also more generally about the roles of tubulin isotypes and kinesin motors in development, and whether there is any specificity between TUBB3 and KIF21A in health and disease.

## Author contributions

DP, BB, and EE researched, wrote, and edited the review. All authors contributed to the article and approved the submitted version.

## Conflict of interest

The authors declare that the research was conducted in the absence of any commercial or financial relationships that could be construed as a potential conflict of interest.

## Publisher’s note

All claims expressed in this article are solely those of the authors and do not necessarily represent those of their affiliated organizations, or those of the publisher, the editors and the reviewers. Any product that may be evaluated in this article, or claim that may be made by its manufacturer, is not guaranteed or endorsed by the publisher.

## Footnotes

^1^ https://www.ncbi.nlm.nih.gov/clinvar

^2^ https://gnomad.broadinstitute.org/

^3^ https://www.ncbi.nlm.nih.gov/clinvar/variation/VCV000058271.2

^4^ https://search.clinicalgenome.org/kb/gene-dosage/HGNC:20772#report_details_haploinsufficiency

^5^ https://search.clinicalgenome.org/kb/gene-dosage?page=1&size=25&search=kif21a

## Abbreviations

AD: autosomal dominant
AF: allele frequency
ASD: autism spectrum disorder
CCDD: congenital cranial dysinnervation disorder
CFEOM: congenital fibrosis of the extraocular muscles
CN: cranial nerve
DN: *de novo*
EOM: extraocular muscle
MCD: malformation of cortical development
MRI: magnetic resonance imaging
NCBI: National Center for Biotechnology Information
PTM: post-translational modification
VUS: variant of uncertain significance
MAP: microtubule associated protein
